# Modeling the factors that influence exposure to SARS‐CoV‐2 on a subway train carriage

**DOI:** 10.1111/ina.12976

**Published:** 2022-02-08

**Authors:** Daniel Miller, Marco‐Felipe King, James Nally, Joseph R. Drodge, Gary I. Reeves, Andrew M. Bate, Henry Cooper, Ursula Dalrymple, Ian Hall, Martín López‐García, Simon T. Parker, Catherine J. Noakes

**Affiliations:** ^1^ Defence Science and Technology Laboratory Salisbury UK; ^2^ School of Civil Engineering University of Leeds Leeds UK; ^3^ Department of Mathematics University of Manchester Manchester UK; ^4^ School of Mathematics University of Leeds Leeds UK

**Keywords:** airborne, close‐range, fomite, SARS‐CoV‐2 modeling, subway, subway train carriage

## Abstract

We propose the Transmission of Virus in Carriages (TVC) model, a computational model which simulates the potential exposure to SARS‐CoV‐2 for passengers traveling in a subway rail system train. This model considers exposure through three different routes: fomites via contact with contaminated surfaces; close‐range exposure, which accounts for aerosol and droplet transmission within 2 m of the infectious source; and airborne exposure via small aerosols which does not rely on being within 2 m distance from the infectious source. Simulations are based on typical subway parameters and the aim of the study is to consider the relative effect of environmental and behavioral factors including prevalence of the virus in the population, number of people traveling, ventilation rate, and mask wearing as well as the effect of model assumptions such as emission rates. Results simulate generally low exposures in most of the scenarios considered, especially under low virus prevalence. Social distancing through reduced loading and high mask‐wearing adherence is predicted to have a noticeable effect on reducing exposure through all routes. The highest predicted doses happen through close‐range exposure, while the fomite route cannot be neglected; exposure through both routes relies on infrequent events involving relatively few individuals. Simulated exposure through the airborne route is more homogeneous across passengers, but is generally lower due to the typically short duration of the trips, mask wearing, and the high ventilation rate within the carriage. The infection risk resulting from exposure is challenging to estimate as it will be influenced by factors such as virus variant and vaccination rates.

**PRACTICAL IMPLICATIONS**

- The prevalence of infection in the passenger community is predicted to have a strong impact on total exposure for passengers; therefore, strategies to minimize the chance of infectious passengers traveling are likely to have a significant impact on overall infection risk for the passenger population.
- The relatively large importance of the close‐range route suggests that strategies to facilitate lower passenger density could have a significant positive impact on infection risk particularly during times when community prevalence of disease is high.
- Mask‐wearing impacts on all transmission routes and therefore strategies to enable high mask‐wearing compliance are predicted to have a noticeable impact on the total dose received by passengers during their trip.
- It is likely that strategies to facilitate hand hygiene for passengers soon after touching high‐touch surfaces will be the most effective approach to decreasing exposure through the fomite route.
- High air change rates combined with short journey times and use of masks reduce the probability of exposure through airborne routes.

## 1 INTRODUCTION

The COVID‐19 pandemic has caused huge economic and societal impacts worldwide, and the suppression of its transmission has become a globally sought and increasingly critical goal as many healthcare systems approach capacity and capability limits. Understanding the locations where transmission happens, the routes of transmission, and the effectiveness of different mitigation measures is important for enabling safer societal interactions. The SARS‐CoV‐2 virus is released in droplets and aerosols carrying the virus during respiratory activities including breathing, talking, and coughing. At close‐range (<2 m) exposure to the full size distribution of aerosols and droplets through inhalation and direct deposition onto mucous membranes is likely. Airborne transmission occurs through inhalation of small aerosols that remain suspended in the air over distances (>2 m). Fomite transmission may also occur through contact with contaminated surfaces and subsequent touching of mucous membranes. The relative contribution of these different routes is unknown, however, risk factors that have been identified include duration of time spent with infected people, close proximity, activities that may generate more aerosol, and enclosed poorly ventilated environments.^1^

There is very little evidence regarding transmission on ground public transport or for conditions that pose a particular risk. In ref. 2 testing and contact tracing among students using school buses in an independent school in Virginia revealed no transmission linked to bus transportation, under universal masking and simple ventilation techniques. An epidemiological study examining long distance rail journeys in China suggested transmission risk was greatest at close proximity, but this may have been influenced by family groups traveling together.^3^ Two outbreak investigations following long duration bus journeys in China concluded that aerosol transmission was a likely explanation, although acknowledged that fomites could have contributed to some cases.^4^, ^5^ Contemporaneous travel by family groups (and so effectively extending duration and nature of within household mixing) and co‐workers (effectively increasing within work mixing) may cause cross‐transmission events where the nature of travel is incidental to the event rather than causation. Such events are not explicitly considered in our work here. Sampling of surfaces and air in buses and subway trains in Barcelona found small traces of SARS‐CoV‐2 RNA (ie, not necessarily the infectious virus) in 30/82 samples, with more positive samples from surfaces than the air and in buses compared to trains.^6^ However, studies on buses in Abruzzo, Italy^7^, and on the London Underground^8^ found no trace of viral RNA in the air or on surfaces. To date, there is no evidence that public transport is a major driver for the pandemic, but as a shared enclosed setting where people may be at close proximity transmission is possible and understanding the factors that influence the likelihood of transmission is important for introducing and managing effective mitigation strategies. This is particularly important as public transport is a necessity for many people, and it can be an environment where social distancing is difficult to maintain, particularly in dense urban transport systems.

Quantitative Microbial Risk Assessment (QMRA) methodologies are one approach to being able to evaluate the potential factors that influence transmission. QMRA is a well‐established methodology for assessing infection risks, using probabilistic modeling to estimate exposure to pathogens through particular routes, coupled with data on dose response to calculate risk.^9^ Models are based upon the physical mechanisms for transmission and therefore can consider the effect of environmental and behavioral interventions at a local scale. A number of modeling approaches have been developed recently which focus on airborne transmission for SARS‐CoV‐2.^10^, ^11^, ^12^, ^13^, ^14^ Close‐range transmission has also been considered in some detail through physics‐based models,^15^, ^16^, ^17^ although the application of these models to quantifying transmission risk has only been used in a small number of cases. The transmission potential through the fomite route has had less attention, but has been modeled for a number of pathogens in the past,^18^, ^19^, ^20^, ^21^ and these methodologies have been recently implemented for SARS‐CoV‐2.^22^ However, few attempts have been made to account for all transmission routes simultaneously when assessing level of exposure in different scenarios.^23^ The role played by each possible transmission route is likely to vary across different environments, and few QMRA models have been developed to quantify the importance of the different transmission routes in these different settings. One SARS‐CoV‐2 example is the work in Azimi et al.^24^, which models and quantifies COVID‐19 transmission, through different routes, for the Diamond Princess cruise ship outbreak and concludes that aerosols may be responsible for 50% of the transmission. The work in ref. ^25^ follows a similar multi‐route QMRA‐based modeling approach when studying SARS‐CoV‐2 transmission mechanisms in a healthcare setting and suggests inhalation (close range and far field) is the dominant exposure route. A small number of studies have also applied multi‐route QMRA models to other respiratory pathogens including^26^ who consider the relative importance of transmission routes for influenza,^27^ who model influenza, norovirus, and SARS transmission on an aircraft,^28^ who consider long‐range airborne and fomite transmission in a hospital outbreak of SARS‐CoV‐1, and^29^ who models a hospital outbreak of MERS‐CoV in Korea. The later two models all focus on real outbreak settings and demonstrate the potential for both airborne and fomite transmission to be important.

In this work, our interest is in developing a QMRA‐based methodology to evaluate exposure to SARS‐CoV‐2 through different routes in a public transport scenario. Although our results here focus on a subway carriage, our methodology is partially based on the work in ref. 27 which was designed for an aircraft cabin and is general enough to be adapted to other public transport scenarios such as a bus or a train. This Transmission of Virus in Carriages (TVC) model estimates exposure to SARS‐CoV‐2 for passengers traveling within a carriage over a single journey and allows one to carry out a comprehensive sensitivity analysis on key parameters. The main aim of our study is to consider the relative importance of different factors which the transport company controls (ventilation, passenger density), and individual controls (mask wearing and hand hygiene), and national policy controls (prevalence). In particular, we evaluate the impact of infectious disease prevalence among passengers, system loading levels, mask‐wearing compliance, and ventilation on the total predicted exposure for passengers. In adapting the original model by Lei et al.,^27^ we have developed a number of new aspects including an approach to model the transient journeys with realistic boarding and alighting behavior seen on subway systems, the attribution of proximal passengers according to area available rather than fixed locations to account for standing on subway services, the use of a range of different droplet models and the application of SAR‐CoV‐2 specific parameters.

In Section 2, we present the main assumptions behind the TVC model, outlining the way in which we model the three different transmission routes (fomite, close‐range, and long‐range airborne), and how the journeys of individual passengers are simulated. In Section 3, we carry out a comprehensive sensitivity analysis on key parameters, reporting our findings on the contribution of each transmission route, and the impact of mitigation. Finally, a discussion follows in Section 4.

## 2 METHODOLOGY

The TVC model is based on the stochastic risk model of Lei et al.,^27^ which was originally developed to quantify the infection risk (by the three different transmission routes) for passengers in an aircraft cabin, for influenza A H1N1, SARS‐CoV‐1, and norovirus. In the TVC model, the exposure of passengers to SARS‐CoV‐2 in a given carriage within an underground system is estimated by considering a number of characteristics of this carriage, such as its internal volume, surface area, and air change rate.

Passengers in the TVC model are individual entities (*agents*) that can board or alight the carriage at any station. Their boarding and alighting behaviors are based upon typical traveling patterns derived from historical London Underground data, as briefly described in Subsection 2.1. Exposure to SARS‐CoV‐2 for susceptible passengers occurs due to infectious passengers traveling on the carriage, depending on the prevalence parameter, ϕ, which is the percentage of passengers traveling who are infectious. Infectious passengers have a fixed viral load ω, and exhale droplets/aerosols of different sizes according to a droplet model. A number of existing exhaled droplet models have been considered in the TVC model and are explored in our results in Section 3: the BLO model,^30^ the Duguid model,^31^ and the Loudon & Roberts (L&R) model,^32^, ^33^ accounting for different respiratory behaviors of the infectious passenger (breathing, speaking, and coughing); see the Supplementary Material for details.

**FIGURE 1:**
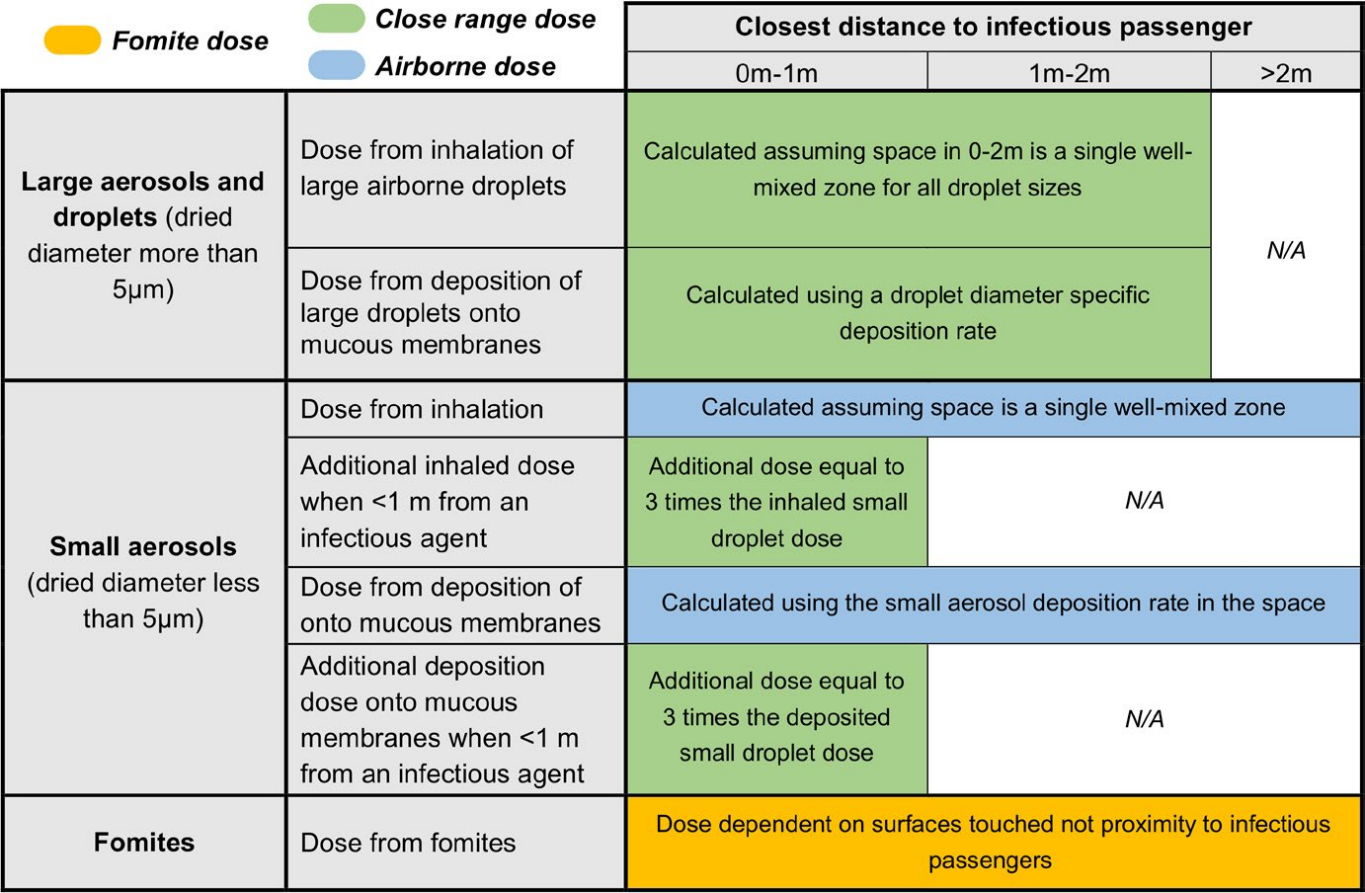
Overview of SARS‐CoV‐2 exposure routes for any passenger in the TVC model. See the Supplementary Material for considerations on the size and evaporation of respiratory droplets. Dose via small aerosols due to background contamination levels on the carriage (i.e., from the well‐mixed single zone model, not related to distance) are considered within the airborne route, while the additional contribution in small aerosol dose due to proximity is considered within the close‐range route, since it is dependent on distance

The TVC model accounts for three routes of transmission: the fomite, the close‐range, and the long‐range airborne routes. Fomite transmission can occur through contact with (potentially contaminated) surfaces representative of those easily contactable within a subway train carriage (primarily handrails and seat rests) during the boarding and alighting process. Once in the carriage, the precise position of each individual is not explicitly tracked, to reduce model complexity, but the likely distance of each passenger (within 1 m, 1 m to 2 m, or beyond 2 m) to an infectious passenger (if present) is estimated based on passenger density at any given time; specific details about this can be found in the Supplementary Material. Close‐range transmission occurs for passengers within 2 m of an infectious passenger.^27^ On the other hand, contamination of the air by small aerosols due to an infectious passenger acting as a source is assumed to be homogeneous across the carriage (well‐mixed space), except for the closest area to an infectious passenger, where passengers within 1 m receive four times as much small aerosol exposure.^27^, ^34^ The TVC model estimates exposure during passengers’ trips on the carriage itself, and not elsewhere (eg, we do not model exposure in the station). A brief summary of these assumptions is given in Figure 1.

### 2.1 Carriages and traveling patterns

The TVC model assumes a carriage with internal volume 53.2 m^3^ and a height 2.148 m; a comprehensive list of parameter values within the TVC model, and their source, can be found in the Supplementary Material. The passenger boarding and alighting behaviors have been generated by using an existing in‐house agent‐based computational model that simulates individuals’ journeys between stations. Agents traverse the 1D tunnel network by following directions to their assigned waypoints in sequence. Agents will board and alight train carriages, assuming there is space available, when located at platforms to continue their journey. As well as station entrances, agents may start and end their journeys on adjoining line trains. Waypoint input data for the model is based on agglomerated historical London Underground data from 2015 for passenger boarding and alighting behavior between 8am and 10am on a weekday. This aims to represent realistic loading patterns over the course of a journey rather than simulate exact travel patterns. In this computational model, 824 different carriage journeys were generated (NB 408, SB 416) which traverse the entirety of a subway line that passes from suburb to suburb via a city center. Variability within these carriages includes variability in both departure time and the carriage's specific location within the train (trains comprise several carriages with different seating and floor areas, as well as location within the train).

**FIGURE 2:**
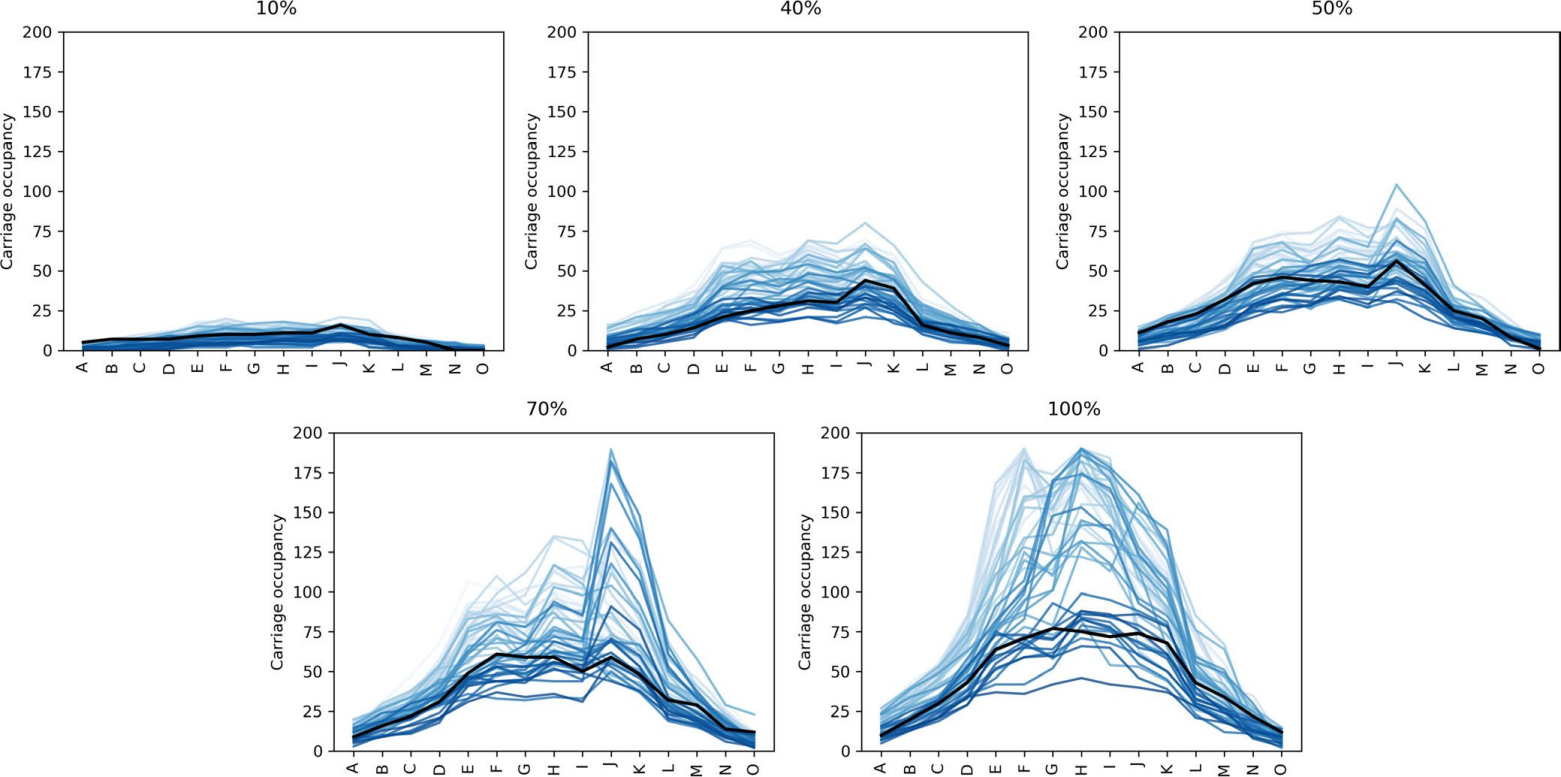
Plots show carriage occupancy against station for system loading percentages of $\rho \in \left\{10\text{\%}\;,40\text{\%}\;,50\text{\%}\;,70\text{\%}\;,100\text{\%}\right\}$. Each line represents a carriage on a single southbound journey between stations A and O with the black line indicating the selected carriage used in the model. Passenger numbers traveling for each loading percentage, during a single journey of the representative carriage, are 28 (10%), 77 (40%), 112 (50%), 132 (70%), and 176 (100%). Light colors indicate trips earlier in time. We observe that the selected carriage is representative of the SB carriages as a whole

Instead of considering different carriage journeys as a stochastic element in the TVC model, which would be computationally prohibitive, a single carriage was selected, to represent an average or representative behavior, for our numerical results in Section 3. This representative carriage,^35^ is depicted as a solid black line in Figure 2. We plot in Figure 2 the visual inspection of the route against stops traveled, highlighting as a black solid line the selected carriage used in the model. A system loading percentage parameter ρ is varied between 10% and 100%, where 100% represents 2015 patronage levels. The total number of simulated passengers per model run is included in the caption for Figure 2 for each system loading value. This total number of passengers per carriage journey is not equal to the carriage occupancy at any given time and does not scale directly with the system loading because carriage loading patterns are influenced by system effects and the selection of representative carriages. The process used to select representative carriage boarding and alighting patterns is detailed in the Supplementary Material. It can be seen that the representative values fall in the center of the range of occupancy values and do not reach maximum carriage capacity values. As highlighted in Figure 2, peak carriage occupancy for some carriages can be double the representative carriage occupancy considered in the model. The loading percentage parameter is then used to scale down the number of passengers within any given route to represent a reduction in public transport usage with respect to pre‐pandemic levels. This parameter is varied in the numerical results in Section 3, to analyze the impact that it can have on our estimates for SARS‐CoV‐2 exposure.

### 2.2 Fomite route

The fomite route consists of dose contributions due to hand‐surface and hand‐mucosal membranes contacts. Surfaces considered within the carriage are selected as those easily contactable by passengers. ^35^ In particular, $N_{T}=114$ non‐porous surfaces are considered which account for grab poles, door handles, horizontal railings, and chair hand rests; see Table 2 in the Supplementary Material. Each passenger touches a fixed number of randomly selected surfaces per journey, one set of $N_{\mathrm{HS}}$ (number of hand to surface touches) surfaces on boarding and another set of $N_{\mathrm{HS}}$ surfaces on alighting. The probability of touching each surface is assumed identical, and the model does not allocate surfaces to particular areas of the carriage, to reflect that passengers have a large degree of freedom over which surfaces they touch when traversing the carriage. Surfaces are assumed to be clean (not contaminated) at the beginning of the carriage trip and no cleaning during the trip is included within the model. Hands of infectious individuals are assumed to be contaminated when boarding the carriage (see Table 2 within the Supplementary Material), while susceptible passengers are assumed to board the carriage with clean hands, and only the dominant hand for each passenger is modeled to estimate the fomite exposure.^21^, ^27^, ^36^

Every time a passenger touches a surface, the concentrations on the passenger's hand, $C_{H}$ (measured in virus plaque‐forming units per square meter, $PFU\cdot m^{‐2}$), and on the corresponding surface, $C_{S}$ [$PFU\cdot m^{‐2}$], are updated as follows^18^, ^19^

$$C_{H}=C_{H0}+\frac{A_{\mathrm{HS}}}{A_{P}}\left(\tau_{\mathrm{SH}}\cdot C_{S0}‐\tau_{\mathrm{HS}}\cdot C_{H0}\right),C_{S}=C_{S0}+\frac{A_{\mathrm{HS}}}{A_{S}}\left(\tau_{\mathrm{HS}}\cdot C_{H0}‐\tau_{\mathrm{SH}}\cdot C_{S0}\right),$$

where $C_{H0}$ and $C_{S0}$ refer to the concentrations just before the touch occurs. $A_{\mathrm{HS}}$ [$m^{2}$] is the area of the hand‐surface contact, $A_{P}$ [$m^{2}$] is the area of the palm, $\tau_{\mathrm{HS}}$ and $\tau_{\mathrm{SH}}$ [–] are the transfer efficiencies (from hand to surface and from surface to hand, respectively) during the surface‐hand contact, and $A_{S}$ [$m^{2}$] is the area of the surface. The equations above assume that contamination on hands and surfaces becomes spatially homogeneously distributed immediately after each contact.^18^, ^19^ During each simulation, the individual concentrations on the surfaces, and passenger's hand, are updated after each hand‐surface contact.

As well as transfer of virus via passengers touching surfaces, the concentration on hands and surfaces is modeled to change through viral inactivation and through loading due to the respiratory behavior of infectious passengers (breathing, speaking, or coughing, as described in the Supplementary Material). Loading and viral inactivation on surfaces and hands occur within the TVC model after the set of hand‐surface contacts happen at any station during passengers alighting and boarding, and immediately after the train starts moving to the next station. This is modeled by means of the following differential equation:

$$\frac{dC}{dt}=\Omega ‐\delta C,$$

where C is the concentration (on the hand or surface) [$PFU\cdot m^{‐2}$], Ω is the source strength [$PFU\cdot m^{‐2}\cdot s^{‐1}$] (which depends on the droplet model considered, as described in the Supplementary Material), and δ is the loss through inactivation [$s^{‐1}$]. For δ>0, this equation has the solution

$$C\left(t\right)=\frac{\Omega }{\delta }+\left(C_{0}‐\frac{\Omega }{\delta }\right)e^{‐\delta t},t\geq 0,$$

for a given initial concentration $C_{0}=C\left(0\right)$. The surface concentration source term, which we can denote by $\Omega_{S}$, is given by

$$\Omega_{S}=N_{p}\cdot \frac{A_{\mathrm{proj}}}{A_{\mathrm{depo}}}\cdot \frac{\sum_{j=1}^{M}\Omega_{j}}{A_{S}},$$

where $N_{p}$ is the number of passengers for whom the surface is the last one touched during boarding [‐], $A_{\mathrm{proj}}$ is the projected area of the surface [$m^{2}$], and $A_{\mathrm{depo}}$ is the plane of the horizontal and vertical spread of the exhaled droplets 0.5 m from the infectious passenger [$m^{2}$]. The specific location of infectors within the carriage is not tracked. Thus, we assume in the equation above that the last surface they touch is the one being contaminated by their respiratory behavior during that particular journey section, indicating that they stay in close proximity to this last surface. In the equation above, we sum across all j=1,…,M droplet sizes of the infectious passenger’s close‐range source term $\Omega_{j}$ [$PFU\cdot s^{‐1}$], which depends on the considered droplet model and their assumed respiratory behavior, as described in detail in the Supplementary Material. We note that the equation above is based on the assumption that virus exhaled by infectors are uniformly distributed on the plane of the horizontal and vertical spread of the exhaled droplets 0.5 m from the infectious passenger. Similarly, the hand source term $\Omega_{H}$ [$PFU\cdot m^{‐2}\cdot s^{‐1}$] is given by

$$\Omega_{H}=\frac{\sum_{j=1}^{M}\Omega_{j}}{A_{P}}.$$

In each simulation of the model, and for a given length of time, Δt [s], the hand and surface concentrations are therefore adjusted according to the expressions

$$C_{H}=\frac{\Omega_{H}}{\delta_{H}}+\left(C_{H0}‐\frac{\Omega_{H}}{\delta_{H}}\right)e^{‐\delta_{H}\cdot \Delta t},\;C_{S}=\frac{\Omega_{S}}{\delta_{S}}+\left(C_{S0}‐\frac{\Omega_{S}}{\delta_{S}}\right)e^{‐\delta_{S}\cdot \Delta t},$$

where $\delta_{H}$ and $\delta_{S}$ are the hand and surface viral inactivation rates, respectively. While breathing and speaking respiratory behaviors may lead to contamination both on hands and surfaces, we assume that infectious passengers would cough onto their hands, and therefore consider $\Omega_{S}=0$ for coughing in our numerical results in Section 3.

Once the concentration on hands has been quantified for passengers during their journey, we can estimate their fomite dose. In particular, we assume that passengers touch their mucosal membranes at a rate $\xi_{m}$ [$s^{‐1}$]. For a fixed number $N_{\mathrm{HM}}$ of hand contacts to the mucosal membranes, the dose can be calculated as

$$D\left(N_{\mathrm{HM}}\right)=\sum_{j=0}^{N_{\mathrm{HM}}‐1}A_{\mathrm{HM}}\cdot \tau_{\mathrm{HM}}\cdot C_{H}\left(j\right),$$

where $\tau_{\mathrm{HM}}$ [–] is the transfer efficiency from hand to mucosal membrane, $A_{\mathrm{HM}}$ [$m^{2}$] is the area of the hand‐mucosal membrane contact area, and $C_{H}\left(j\right)$ [$PFU\cdot m^{‐2}$] represents the concentration on the hand after j touches. We note that the concentration on the hands after $N_{\mathrm{HM}}$ touches is governed by the initial concentration, the amount transferred to the membranes and the amount that has decayed on the hands, which is governed by the hand viral inactivation rate $\delta_{H}$. When estimating the fomite dose, we assume that passengers do not touch their mucosal membranes during their journeys, but that they will touch them after alighting for an amount of time $T_{a}$ [s]. This assumption represents social pressure expected to dissuade face‐touching during the journey, and since in our simulations in Section 3, a sub‐set of passengers may be wearing masks. After alighting, it is likely that passengers will remove their masks if allowed and be able to touch their faces. The time available for transfer via hand‐mucosal membranes contacts would depend in practice on how long the passenger takes to clean their hands, which relates here to the parameter $T_{a}$.

In practice, and for a given rate $\xi_{m}$ [$s^{‐1}$] of membrane touches, the fomite dose above is computed by updating the hand and mucosal membrane concentrations after each touch of the mucosal membranes by means of sequentially implementing the following steps:

$$C_{H}\leftarrow C_{H}\cdot e^{‐\frac{\delta_{H}}{\xi_{m}}}\}\;\text{viral decay on hands between hand - mucosal membranes contacts}$$

$$\left.\begin{array}{c} C_{M}\leftarrow C_{M}+C_{H}\cdot \tau_{\mathrm{HM}}\cdot \frac{A_{\mathrm{HM}}}{A_{M}}\\ C_{H}\leftarrow C_{H}\left(1‐\tau_{\mathrm{HM}}\cdot \frac{A_{\mathrm{HM}}}{A_{P}}\right) \end{array}\right\}\text{viral transfer during hand - mucosa contacts}$$

and where the total number $N_{\mathrm{HM}}$ of mucosal touches after alighting is estimated by multiplying the time after alighting parameter $T_{a}$ by the touches per second parameter $\xi_{m}$ and rounding down. We note that $\xi_{m}^{‐1}$ [s] represents the average time between membrane touches.

### 2.3 Long‐range airborne route

Passengers sharing the carriage with an infected person are assumed to be exposed to virus through a long‐range airborne route due to aerosols less than 5 μm (dried) in diameter; the initial diameter is assumed to be up to 20 μm with evaporation to 25% based on.^33^ This is modeled here through a state‐space implementation^37^ of a single zone model, assuming the air in the carriage is fully mixed. The concentration of virus in the air within the carriage volume V at time t+Δt is given by [Parker et al.^37^, Eq. (20)]

$$C_{\mathrm{LR}}\left(t+\Delta t\right)=C_{\mathrm{LR}}\left(t\right)e^{‐R\cdot \Delta t}+\left(1‐e^{‐R\cdot \Delta t}\right)\frac{u}{V\cdot R},$$

where u [$PFU\cdot s^{‐1}$] is a source of virus (from infectious passengers), $C_{\mathrm{LR}}\left(t\right)$ is the airborne concentration in the carriage at time t and $R=\frac{r_{v}}{V}+r_{i}+r_{d}$ [$s^{‐1}$] is the combination of all removal rates: ventilation ($r_{v}$), viral inactivation ($r_{i}$), and deposition ($r_{d}$), where $r_{v}$ is the fresh flow rate and V is the volume. The long‐range airborne exposure during the time period Δt, $E_{\mathrm{LR}}\left(t,t+\Delta t\right)$ [$PFU\cdot m^{‐3}\cdot s$], is then modeled as [Ref. 37, Eq. (23)]

$$E_{\mathrm{LR}}\left(t,t+\Delta t\right)=\frac{\left(1‐e^{‐R\cdot \Delta t}\right)\left(V\cdot C_{\mathrm{LR}}\left(t\right)‐\frac{u}{R}\right)+\Delta t\cdot u}{V\cdot R}.$$

We note that, once an exposure is calculated for any time t, the corresponding inhaled dose up to time t is given by $D_{\mathrm{LR}}\left(t\right)=E_{\mathrm{LR}}\left(0,t\right)\cdot BR\cdot RF$, where BR [$m^{3}\cdot s^{‐1}$] is the breathing rate of the passenger and RF [–] is the retained fraction of virus that deposits in the respiratory tract and assumed to be different between small aerosols and large droplets. The airborne dose for each passenger is calculated upon arrival at every station, and before the carriage viral airborne concentration is updated for the next segment of the carriage journey. The source term u is calculated by summing up the individual sources corresponding to each infectious passenger on that segment of the trip. We note that the dose obtained by direct deposition of small aerosols onto mucosal membranes is also computed, by multiplying the exposure by the deposition rate, the carriage volume, and by the ratio between the mucosal membranes area with respect to the total available deposition area. However, as the area of each passengers, mucosal membranes are relatively small to the total surface area within the carriage, and the deposition rate is also small, the dose by direct small aerosol deposition ends up being <0.1% of the total small droplet dose, where most of the contribution is from inhalation.

### 2.4 Close‐range route

In order to estimate the close‐range (<2 m) dose that passengers receive by being in close proximity to an infectious passenger, we follow here the approach in Lei et al.^27^ In particular, this dose is decomposed into:

(i) A factor 4 increase (with respect to that predicted in Subsection 2.3) in the exposure the passenger receives due to inhalation of small aerosols (for passengers within 1 m only), see ref. 27, Eq. (1)] and Figure 1. The assumption is that the number of small droplets per unit volume is approximately uniform outside of 1 m , but increases linearly inside of 1 m with decreasing distance to the infectious passenger. In our computations, passengers within 1 m are assumed to be at a representative distance of 0.5 m away from the infectious passenger.
(ii) Dose received as a result of direct deposition of large droplets directly onto mucosal membranes.
(iii) Exposure to an additional airborne concentration via inhalation occurring from large aerosols initial diameter >20 μm that typically deposit by 2 m from the source.

The dose contribution in (i) above can be directly computed by scaling the long‐range airborne dose computed in Subsection 2.3, just for passengers within 1 m of an infectious passenger at any given time during their trips. For the additional dose related to large droplets (contributions (ii) and (iii)), and for each droplet size (see the Supplementary Material), the TVC model calculates the related airborne concentration around each infectious passenger as the train moves through the journey using the state‐space approach in Subsection 2.3, but using the vertical cylindrical volume within 2 m of the infectious passenger as the zone volume. In addition, for a given droplet size j=1,…,M, $k_{d}\cdot {\left(\frac{d}{2}\right)}^{2}$ [$s^{‐1}$] is its deposition rate (d being the dried droplet diameter [m] and $k_{d}$ the coefficient of particle deposition [$s^{‐1}\cdot m^{‐2}$]) and the source term is specific to the large droplet diameter, following the approach in ref. 27. Then, for a given exposure $E_{d}\left(t\right)$ [$PFU\cdot m^{‐3}\cdot s$] due to deposition of large droplets of diameter d, the corresponding dose is calculated as $E_{d}\left(t\right)\cdot k_{d}\cdot {\left(\frac{d}{2}\right)}^{2}\cdot V_{2m}\cdot \frac{A_{M}}{A_{2m}}$. Here, $V_{2m}$ is the volume of the carriage within 2 m of the infectious passenger, and $A_{2m}$ is the surface area within 2 m of the infectious passenger; see the Supplementary Material for further information.

### 2.5 Default parameter values and assumptions

Table 1 shows a list of default key parameter values used within all current simulations unless otherwise specified in figure captions. A comprehensive list of all the parameter values considered within the TVC model is provided in Table 2 within the Supplementary Material. All passengers and trips are considered within the results, including those where no infectious passenger was present in the carriage. Dose values are given in number of virus plaque‐forming units (PFU) for a specific infectious viral load ($\omega =3.61\times 10^{12}PFU\cdot m^{‐3}$ in respiratory fluid refs. 38, 39), and we also include all fractional values since these would correspond to multiple viruses for a higher viral load. As we describe in Subsection 3.5, the source droplet model has a significant effect on the absolute dose received, where Duguid^31^ and Loudon & Roberts (L&R)^32^, ^33^ models typically predict a higher value than the BLO model.^30^ Hence, when interpreting these results we focus on qualitative trends and relative behaviors rather than referring to specific absolute numbers of virions. As a dose‐response curve for SARS‐CoV‐2 is not available for inoculation via different means, we will focus on analyzing received doses rather than infection probabilities. However, we do discuss in Section 4 how one could estimate risk of infection using a dose‐response curve such as that for HCoV‐229E,^40^ if doses from the fomite, close‐range, and long‐range airborne routes could be simply summed when implementing the dose‐response function.

**TABLE 1:** Default parameters used in the simulations. For each parameter combination, 500 simulations were run for the results in Section 3

Parameter name	Default Value	Range	Parameter name	Default value	Range
Fresh‐air changes per hour (ACh^−1^):	127	1, 4, 13, 40 and 127	Mask‐wearing proportion:	75%	0%, 75%, 90% and 100%
Prevalence:	1%	0.02%, 0.1%, 1% and 2%	Droplet model:	BLO coughing	BLO breathing, BLO coughing, BLO speaking, Duguid, L&R
Passenger loading:	50%	10%, 40%, 50%, 70% and 100%			

## 3 RESULTS

**FIGURE 3:**
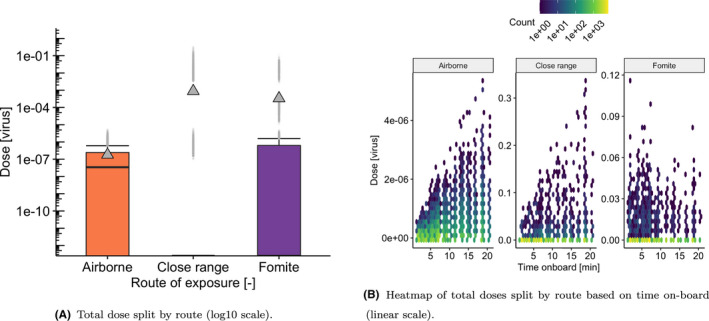
Dose inoculated to susceptible passengers split by route of transmission for the BLO droplet model, 50% loading, 1% prevalence, 127 ACh^−1^ and 75% mask compliance

Our results first look at overall exposure predictions based on default parameter values listed in Table 1, and then, we look at the effect of changing particular parameter values on these predictions. For all boxplots presented in this Section, the central line shows the median value while the box represents the interquartile range and whiskers show 1.5x interquartile range, while points show outliers either side of this value. Triangles show mean values. Doses equal to zero are set to 10^–16^ for the purpose of constructing the boxplots, falling below the limits of the vertical scale. Figure 3 represents the total dose received by passengers split by route of inoculation (airborne, close‐range, and fomite) in the form of boxplots (Figure 3A) as well as a heatmap (Figure 3B) of time on‐board vs dose received, based on default parameter settings (1% prevalence, 75% mask wearing, 50% loading, 127 ACh^−1^ and using the BLO coughing model). The default air change rate is based on an assumed supply rate of 10 L/s/person for theoretical crush capacity within the carriages.^35^ Figure 3A shows that when considering the mean dose, close‐range exposure dominates, followed by fomite and long‐range airborne routes, which are one and three orders of magnitude lower, respectively. However, the median values reflect a different picture, where airborne dose is higher than both the close‐range and the fomite doses (3.5 x 10^–8^ vs. 0). Time on‐board is seen to be an important factor for both airborne and close‐range routes, while the fomite route is less dependent (see Figure 3B). At the 1% prevalence level, the number of 0‐doses dominates the median values suggesting that the mean values are heavily affected by infrequent or *opportunistic* events, represented by the outliers, particularly for the close‐range and the fomite routes. These opportunistic events are directly related to specific individuals being at close proximity to infectious passengers during their trip (contributing to their close‐range exposure), or by chance touching particularly contaminated surfaces during boarding or alighting (contributing to their fomite exposure).

In the following Subsections, we use boxplots to explore the sensitivity of our exposure predictions to the key parameters in Table 1, while keeping all other parameters fixed. These boxplots show the distribution of doses over all model runs. However, some interpretations and insights from these numerical results arise from looking at particular model runs, which are depicted by “waffle” plots. These waffle plots represent the computed dose for all passengers within a single simulation/trip while depicting the traveling patterns for each passenger (ie, origin, destination, and sub‐set of the trip shared with an infectious passenger). The journeys shown in these waffle plots have been chosen at random and can be seen as illustrative. In these plots, infectious passengers are shown colored in orange, while a white‐to‐green gradient is used to represent level of exposure for each non‐infectious individual. Orange lines represent the duration of where an infectious passenger was on‐board. Note that the stations A‐O are presented in chronological order based on Figure 2.

### 3.1 Effect of percentage of infectious passengers (prevalence)

**FIGURE 4:**
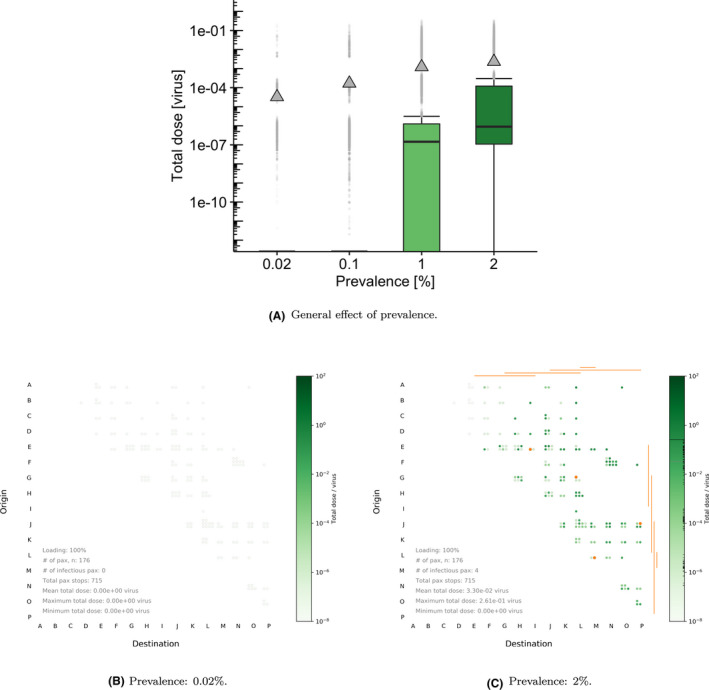
Boxplots and waffle plots comparing the effect of disease prevalence ϕ, for the BLO model. For the boxplots in (A), default parameters are chosen: 127 ACh^–1^ , 50% loading, 1% prevalence, and 75% mask‐wearing compliance. For the waffle plots in (B) and (C), and for illustrative purposes, we select 127 ACh^–1^ , 100% loading, 0% mask wearing and vary the prevalence parameter. Orange lines represent part of the journey where an infectious passenger is on board

Figure 4A shows how the distribution of total doses changes with respect to infection prevalence among the passengers. The impact of the prevalence parameter on the total dose is significant, and higher prevalence values lead to an increase in the dose received, both in terms of the mean and median values. The mean dose shows a linear relationship with prevalence (see Table 2), but the increase in the median value of the dose is not linear, showing an approximately order of magnitude increase when prevalence increases from 1% to 2%. The summary statistics of doses for passengers in these simulations in Table 2 show the median dose is four to five orders of magnitude lower than the mean dose. These results suggest that most passengers receive a zero dose and a small number of passengers receive a much higher dose. The high mean relative to the median values suggests that relatively higher doses for a small number of passengers significantly affect the exposure distribution. These *outliers* can be interpreted as specific individuals involved in *opportunistic events* in these simulations, corresponding to passengers who might have shared some of the trip at close proximity to an infectious individual, or touched particularly contaminated surfaces during their trip. Reductions in the value of the prevalence below 1% allow for the removal of some outliers in the exposure distribution, significantly reducing the mean dose across the population of non‐infectious passengers.

**TABLE 2:** Total dose [PFU] received by non‐infectious passengers depending on the prevalence percentage

Prevalence percentage [0–100]	0.02%	0.1%	1%	2%
Median dose	0	0	1.46E−07	8.98E−07
Mean dose	3.20E−05	1.73E−04	1.23E−03	2.32E−03
Total non‐infectious passengers	55 988	55 929	55 408	54 844
Total non‐zero doses	1133	5752	32 867	44 501
% non‐zero doses	2	10	59	81

Examining a specific model run with different prevalence values allows one to better understand these behaviors. Figure 4B,C show total dose received by non‐infectious passengers in a single trip simulation for the lowest and highest prevalence values. In this particular simulation, for the lowest prevalence value there were no infectious passengers on board, leading to zero exposure. Increasing prevalence up to 2% increases the number of infectious passengers to 4 in this simulation. This would directly increase the number of passengers being at close proximity of an infectious passenger at some time during their trip, and the amount of surfaces becoming contaminated.

### 3.2 Effect of passenger loading with respect to pre‐COVID‐19 values

**FIGURE 5:**
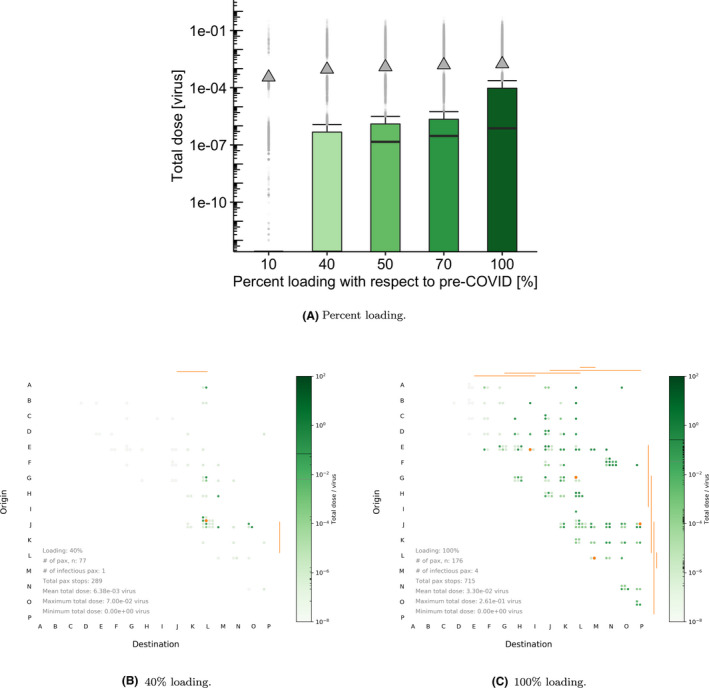
(A) Boxplots of total dose received for the BLO model and default parameter values in Table 2. (B, C) Waffle plots comparing the effect of different passenger loading (ρ) levels with respect to pre‐COVID‐19 numbers. For the waffle plots, and for illustrative purposes, we select 127 ACh^–1^ , 2% prevalence, and 0% mask‐wearing compliance

In Figure 5A and Table 3, we investigate what the impact of varying the system loading percentage is on the total dose exposure. Loading has an important effect on the distribution and median values of the total dose received, with larger values for higher loading, while mean values are less strongly affected by loading. The higher mean relative to the median suggests that high doses for a small number of passengers are important, exemplifying that to all effects these correspond to relatively rare events. It is also worth noting that it is difficult to remove these outliers by decreasing the loading, where even at 10% loading some of these can still be observed where 19% of doses were above the upper whisker. This suggests that in order to decrease the number of those “higher risk” events, joint approaches are needed (ie, in these simulations, there is an interplay between loading, prevalence, and other mitigation strategies such as mask wearing). We also note that the impact of these outliers on the whole distribution effectively decreases as loading increases because more passengers are closer to infectious passengers or come into contact with contaminated surfaces, leading to higher doses for more passengers rather than just a few. Rising from 70% to 100% loading, the median dose increases 60% where we note that the close‐range and fomite routes contribute to the majority of passengers’ exposure (highlighted in Figure 5A). Waffle plots are then used to exemplify a typical passenger journey, where a substantial increase in doses for passengers is seen with destinations in the latter two‐thirds of the route, and when loading increases (Figure 5B vs Figure 5C).

**TABLE 3:** Total dose received by non‐infectious passengers depending on the loading percentage

Loading percentage [0–100]	10%	40%	50%	70%	100%
Median dose	0	0	1.46e−07	2.96e−07	7.40e−07
Mean dose	3.53e−04	9.12e−04	1.23e−03	1.55e−03	1.74e−03
Total non‐infectious passengers	13 854	38 094	55 408	65 307	87 082
Total non‐zero doses	2618	17 137	32 867	42 003	64 317
% non‐zero doses	19	45	59	64	74

### 3.3 Effect of mask‐wearing compliance

**FIGURE 6:**
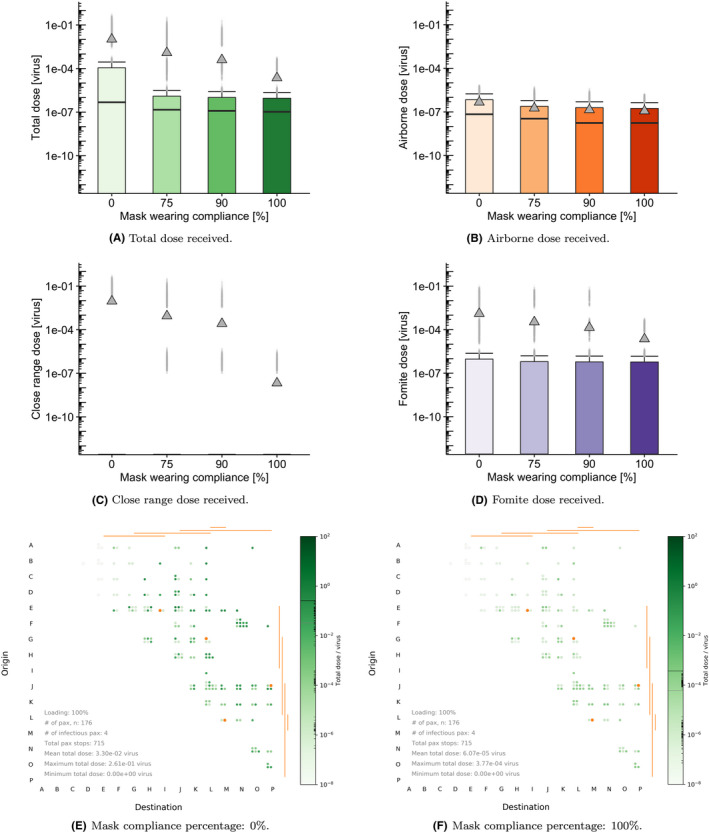
Top rows: Boxplots showing the effect of varying mask‐wearing compliance on (A) the total dose, and the dose for each particular route of exposure: (B) airborne, (C) close‐range, and (D) fomite using default 50% passenger loading, 1% prevalence and 127 ACh^–1^ . Bottom row: waffle plots showing a single train journey at (E) 0% and (F) 100% mask compliance respectively. For the waffle plots, we select 127 ACh^–1^ , 100% passenger loading and 2% prevalence for illustrative purposes

The effect of the proportion of passengers wearing masks was evaluated visually in terms of the dose received by non‐infectious passengers in Figure 6. The main assumptions about the efficacy of masks in the TVC model can be found in the Supplementary Material. Table 4 shows quantitative statistics of the mean and median doses received by non‐infectious passengers depending on the percentage of passengers wearing a mask (including those who might have been infectious). Mask wearing has a modest effect on the median dose, where an approximately 4.7x decrease is observed between 0% and 100% adherence. This is close to what would be expected from the combined effect of 50% reduction on production and inhalation of small aerosol. Increasing the proportion of passengers wearing masks above 75% has little effect on the doses below the 75th percentile of total dose received. However, the mean dose received is strongly dependent on the proportion wearing a mask, and the impact of mask wearing on the outliers can be clearly observed. This is likely to be due to a combination of reduction in source of virus as well as reduction in inhalation (large droplets and small aerosol) and deposition onto mucosal membranes, which significantly decreases the doses related to the opportunistic events, specially for adherence levels near 100%. We note that in reality, some of those outliers representing higher doses might be the ones leading to actual infections and the corresponding generation of transmission chains at the community level.

**TABLE 4:** Total dose received by non‐infectious passengers depending on the proportion of passengers wearing masks

Mask‐wearing percentage	0%	75%	90%	100%
Median dose	4.79e–07	1.46e−07	1.22e−07	1.05e−07
Mean dose	1.04e−02	1.23e−03	4.01e−04	2.30e−05
Total non‐infectious passengers	55 408	55 408	55 408	55 408
Total non‐zero doses	32 867	32 867	32 867	32 867
% non‐zero doses	59	59	59	59

Figure 6 shows the break‐down of the doses received by uninfected passengers split by route of exposure. The close‐range dose shows a scattered pattern among passengers (see Figure 6C), where the pattern results from the randomization of proximity. These are typically the highest doses compared to those from the fomite and airborne routes. Comparing 0% and 100% mask‐wearing compliance shows over 5 orders of magnitude reduction in the close‐range dose received. This is a result of the assumption that 100% large droplets are captured for individuals wearing masks, so that the residual close‐range dose with masking is due to exposure to small aerosol at close range. The airborne doses in Figure 6B show a different pattern, with a more uniform distribution over passengers. The mean values are substantially lower than the close‐range doses at 3–5 orders of magnitude less. Fomite doses (see Figure 6D) are also generally scattered in their pattern among passengers due to the stochastic nature of surface touching, with a wide range of doses received.

### 3.4 Effect of fresh‐flow ventilation rate

**FIGURE 7:**
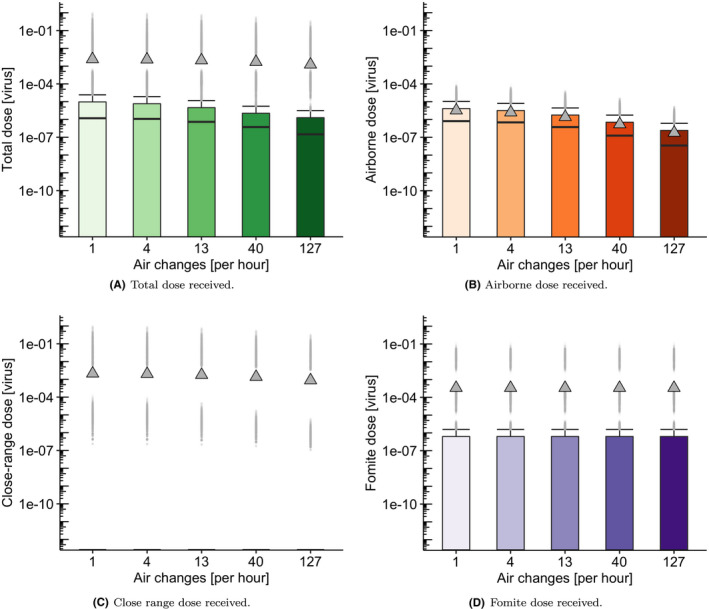
Boxplots showing the effect of varying air change rate on (A) total dose, (B) airborne dose, (C) close‐range dose, and (D) fomite dose. Distributions of dose received by non‐infectious passengers for five different air change rates

The effect of the fresh‐flow ventilation rate is investigated in both airborne and close‐range exposure routes (see Figure 7). Although we explore the impact of reducing the highest ventilation rate on exposure to obtain a qualitative understanding of the corresponding dynamics, we note that the results for lower ventilation rates should be interpreted with caution, since the well‐mixed assumption in Subsection 2.3 might not be appropriate for poorly ventilated scenarios. In those situations, specific areas of the carriage might have significantly higher concentrations than the rest.

Generally speaking, an increase in ventilation rate leads to a decrease in the airborne dose received, both in terms of the mean and median values (see Table 5). However, the reduction does not scale with the inverse of the ventilation rate alone. There are other factors determining the scaling of the dose, for example, additional loss terms representing viral decay or deposition become more important at lower air change rates as do the short occupancy times. The analysis of the total dose shows just a minor effect with increase in ventilation rate. This is expected because the close‐range and fomite routes have a greater contribution to the total dose than the long‐range airborne route for this scenario. Concentrations take longer to build up at lower air change rates relative to the short occupancy times for this scenario. Finally, we note in Figure 7B that mean values corresponding to the long‐range airborne dose are closer to median values than when comparing to the other transmission routes (close‐range and fomite). This seems to indicate that the long‐range exposure by small aerosols would be more homogeneously spread across individuals in the carriage (sharing the trip with an infectious passenger) than the exposure through the close‐range and fomite routes (which are more stochastic and heavily depend on a non‐infectious individual randomly getting close enough to an infectious passenger, or touching particularly contaminated surfaces).

**TABLE 5:** Total dose received by non‐infectious passengers depending on ventilation rate

Fresh‐flow air changes per hour [ACh^−1^]	1	4	13	40	127
Median dose	1.17e−06	1.08e−06	7.33e−07	3.73e−07	1.46e−07
Mean dose	2.46e−03	2.35e−03	2.12e−03	1.72e−03	1.23e−03
Total non‐infectious passengers	55 408	55 408	55 408	55 408	55 408
Total non‐zero doses	32 867	32 867	32 867	32 867	32 867
% non‐zero doses	59	59	59	59	59

### 3.5 Effect of source droplet model

**FIGURE 8:**
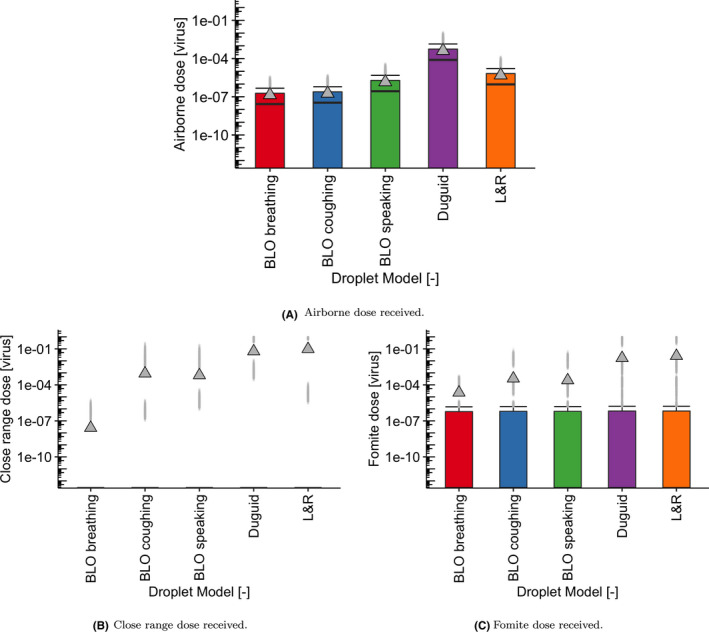
Boxplots showing the effect of the five droplet models using default settings for prevalence (1%), loading (50%), air change rate (127 ACh^–1^ ), and mask‐wearing proportion (75%): on (A) airborne dose, (B) close‐range dose, and (C) fomite dose

**FIGURE 9:**
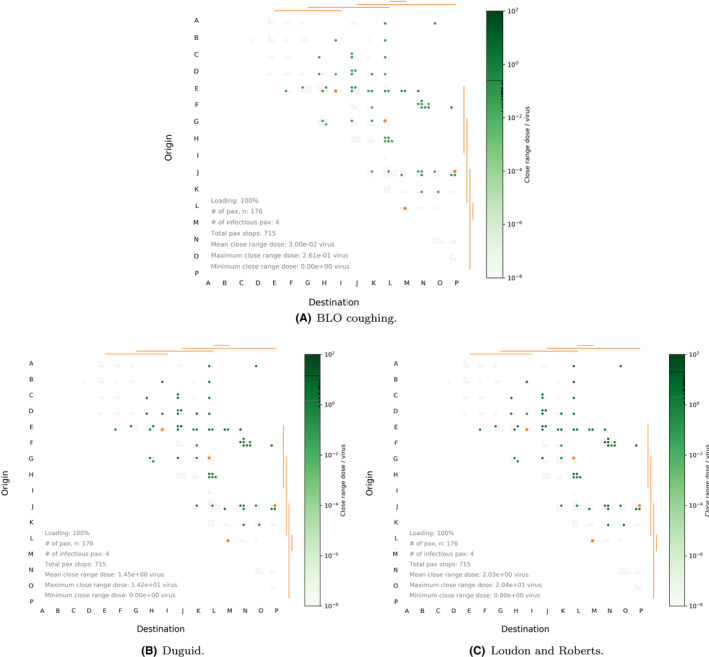
Waffle plots highlighting close‐range dose received under 0% masking, 2% prevalence, 100% loading, and 127 ACh^–1^  air change rate, for the (A) BLO coughing, (B) Duguid, and (C) Loudon and Roberts models

The effect of the source model is shown in Figure 8 where we use default settings for prevalence, loading, air change rate, and mask‐wearing proportion (1%, 50%, 127 ACh^−1^, 75%). It is important to note that there is a large difference between the doses estimated by the different source models rendering the close‐range doses the most variable between models. To illustrate this, we plot the predicted close‐range doses corresponding to the unmasked scenario in Figure 9 showing the effect of using Duguid^31^ or Loudon & Roberts^32^, ^33^ vs the default BLO coughing model.^30^ Historical experimental results^31^, ^32^, ^33^ correspond to nearly two orders of magnitude increase over data from the fitted BLO models^30^ included in the plots above.

It is also important to note that while a single point value for the viral load of $\omega =3.61\cdot 10^{12}\;PFU\cdot m^{‐3}$ has been used as input for these simulations, heterogeneity in this value is expected across different infectious individuals, and that considering a different value would lead to different estimates in terms of absolute number of virions for the predicted doses. As a result, our interpretation of results above is based on exploring relative behaviors and the impact of key parameters or mitigation strategies, rather than commenting on particular absolute numbers of virions. Importantly, when looking at the interaction between the variation in key parameters in the previous Subsections, and the effect of the droplet model, we find that regardless of the droplet model used, the same trends as discussed in the previous sections are borne out. Thus, our conclusions on the relative impact of key parameters on exposure are not affected by the particular droplet model under consideration, or the particular viral load considered; see Figures 4 and 5 within the Supplementary Material.

## 4 DISCUSSION

We have presented the TVC model, which predicts SARS‐CoV‐2 exposure through three routes (close‐range, long‐range airborne, and fomite) in a subway train carriage under a variety of conditions. In particular, we have explored different infection prevalence levels in the traveling population, percentage passenger loading, mask‐wearing compliance, and ventilation rates.

Results suggest that higher disease prevalence levels lead to an increase in the dose received through all routes both in terms of the mean and median values, due to the higher probability that an infectious passenger is traveling. The increase in the median value of the dose is not linear with prevalence, showing approximately one order of magnitude increase when prevalence increases from 1% to 2%. It is also observed how reductions in the value of the prevalence below 1% seem to allow for the removal of some outliers (higher risk opportunistic events) in the exposure distribution, significantly reducing the mean dose across the population of non‐infectious passengers. Our results suggest an interplay between loading levels and prevalence values, where the actual absolute number of infectious passengers traveling jointly depends on both parameters. Higher values of prevalence and loading would lead to the riskiest scenarios.

Comparison of mean doses indicates fomite doses tend to be an order of magnitude lower than their close‐range counterparts, and that the long‐range airborne dose is several orders of magnitude lower. This large difference can be explained in part by the high air change rate and the relatively short duration of shared trips between non‐infectious and infectious passengers in this scenario. We note that the single zone well‐mixed assumption might be particularly well‐suited for higher ventilation rates such as those representative of some London Underground Lines. Smaller air change rates or larger carriages could require more complex computational fluid dynamics, eddy‐diffusion^41^, or zonal airflow approaches in order to obtain higher resolution airborne exposure estimates; we acknowledge that the TVC model may result in an underestimate of airborne doses for some passengers at the lowest ventilation rates. We also acknowledge that under low ventilation rates, or in alternative public transport settings, the additional ventilation provided by the doors opening during stops could have an effect and could potentially be incorporated into the model. Similarly, we assume the source is isotropic (under the well‐mixed hypothesis) in that orientation of the infectious passengers can be ignored such as in the case laid out by.^27^

Consideration of the distribution of doses is also important. Both fomite and close‐range doses have a median value that is much lower than the mean, suggesting that both significantly depend on infrequent events—traveling at close proximity of an infectious passenger, or touching a heavily contaminated surface during boarding or alighting. On the other hand, the predicted long‐range airborne dose is significantly more homogeneous across the population of passengers with mean and median values closer to each other and less outliers. Although the airborne and total exposure is low in our underground train carriage scenario, there may be other indoor scenarios (eg, infectious individual with a significantly higher than usual viral load sharing a poorly ventilated space with a large number of individuals for a significant amount of time) where this homogeneous distribution of doses leads to a high enough median dose to result in many infections. On the other hand, a particularly high viral load would only significantly increase some individual exposures through opportunistic events for the close‐range and fomite routes, and hence, the number of infections caused would be more limited.

We also note that the scenario considered here has certain features that could favor close‐range and fomite exposures, relative to longer‐range exposure, as our results suggest. Passengers generally travel for short periods of time in a highly ventilated setting, with reasonable chances of coming into close proximity of each other and touching a small sub‐set of surfaces contacted by a significant amount of people. Our results then suggest that in a scenario like this one, the close‐range route might be the most significant one, and that exposure through the fomite route cannot be neglected. The fomite dose heavily relies on opportunistic events, yet this can be relatively easily mitigated through facilitating hand hygiene for passengers during and after their trip and by passengers avoiding touching their face during a trip. We note that in the TVC model, frequently touched or easily contactable surfaces within the carriage are treated identically, and have an equal probability of contact for each passenger during boarding and alighting. Although the model may as a result not capture the importance of specific very high touch surfaces, this assumption is sensible to represent that passengers can move relatively freely across the carriage in an underground rail system. However, this approach might not be well‐suited for other public transport settings such as buses, long‐range trains, or planes, and a more complex representation of hand‐surface contact behaviors may be required in those alternative scenarios.

The relatively large importance of the close‐range route suggests that strategies to facilitate social distancing can have a significant impact on infection risk. This would directly depend on prevalence and controlling loading levels accordingly. Close‐range exposure is also predicted to be significantly reduced through increased mask‐wearing adherence. The prevalence of infection in the passengers’ community is predicted to have a strong impact on total exposure and while the prevalence at the population level is difficult to control, our results suggest that any strategies to prevent infectious people from traveling could be one of the most effective strategies to decrease overall infection risk for the traveling population. We note here that the prevalence parameter (defined as the percentage of passengers who are infectious) needs to be interpreted cautiously, since the prevalence of the disease within the passengers population might well differ from the prevalence of infection in the community.

As with all models, there are a number of limitations and assumptions that may influence the model outcomes. Due to the large number of parameters involved in the TVC model, many of the parameters have been fixed to specific values. While reliable estimates for some parameters are available, as described in Table 2 within the Supplementary Material, there are some which have been assumed according to the existing knowledge at the moment, and which could be adjusted once more information becomes available. In particular, precisely estimating behavior‐related parameters such as the number of surfaces touched by passengers during boarding or alighting, or the time for passengers to sanitize their hands after alighting, would require detailed CCTV or similar data for a particular public transport setting. This has been successfully implemented for QMRA approaches in other environments.^42^ The results in Section 3.5 have highlighted the dependence on the data used for the droplet source model and the respiratory activity. There is a clear need for better data on respiratory source terms across all droplet sizes as well as behavioral information on respiratory activity in public spaces. The model is run for a representative viral load based on^38^ and assumes a relationship between RNA copies and PFU,^39^ and that virus is distributed evenly by volume in respiratory particles. A small number of people may have a viral load that is 2–3 orders of magnitude higher,^38^ which would proportionally increase the doses predicted by all routes in the model. Similarly, if the relationship between RNA and infectious virus varies by particle size or transmission route, this would also alter the predicted exposure. At this time, these and other viral emission parameters remain unknown, and hence, our model is based on the best evidence at the time.

A dose‐response curve for SARS‐CoV‐2 is not yet available, and furthermore, the contribution of each dose (ie, each route of exposure) to individual infection risk may still be unclear even if and when it is obtained.^24^, ^27^ Consequently, we have analyzed the results from the TVC model based on relative exposures and qualitative trends to try and understand the effect of key parameters and mitigation strategies. In this model, we do not split the upper and lower respiratory tract dose contributions,^27^ and direct computation of infection risk, which could allow for comparisons with other multi‐route models such as the one in ref. 24 for the outbreak on the Diamond Princess cruise ship, is complex. If one were to assume in a hypothetical situation that the fomite, close‐range, and airborne doses could be considered comparable and cumulative, one could use the HCoV‐229E exponential dose‐response curve as a way of estimating infection risk in the worst‐case scenario for a comparative pathogen.^40^ Under these hypotheses, risk can then be calculated for different scenarios.

**FIGURE 10:**
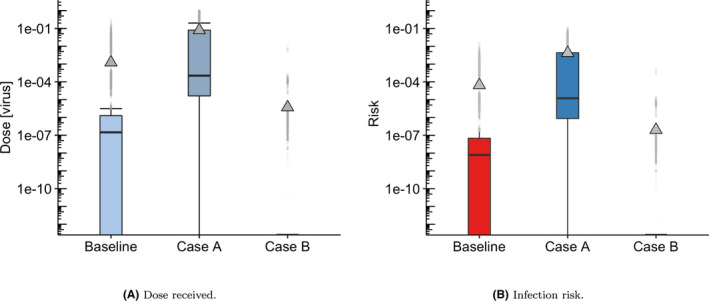
Boxplot showing (A) Dose received and (B) Infection risk (ie, individual probability of infection for each predicted dose) in three different scenarios, using the HCoV‐229E exponential dose‐response curve^40^

In Figure 10, three scenarios in terms of key parameter values are considered for illustrative purposes: Baseline = (1% prevalence, 50% loading, 127 ACh^−1^, 75% mask wearing), Case A = (2% prevalence, 100% loading, 1 ACh^−1^, 0% mask wearing), and Case B = (0.02% prevalence, 100% loading, 127 ACh^−1^, 90% mask wearing). In general, the mean infection risk probability is significantly higher than the upper quartile, alluding to the hypothesis that a few passengers may become infected related to opportunistic or rare events under these circumstances. Using a Bernoulli distribution with either a 1 or a 0 response, representing an infection or not from each one of the predicted exposure doses and corresponding individual infection risk probabilities, we can predict the number of passengers infected per 1000 passenger journeys. From the individual risks predicted under the baseline assumptions of 1% prevalence, 50% loading, 127 ACh^−1^ and 75% mask wearing, we see that 1 person is likely to become infected with another 1 possible based on the mean and standard deviations obtained from 1000 Bernoulli simulation runs. Under the worst case scenario (Case A) which could be roughly interpreted as a situation with very high prevalence among passengers, no mitigation in place and very low ventilation, this mean increases to 5 per 1000 with a standard deviation of 10 infections. Case B represents a potentially realistic *return to normality* scenario with 0.02% prevalence, 100% loading, 127 ACh^−1^ and 90% mask wearing. In this situation, and using the current viral emission rate, the mean infection rate per 1000 passengers would be 0 with a standard deviation of 0. However, if only 75% of people wore masks this would increase to 1 likely infection (and 1 possible) per 1000. Under a ventilation failure (1 ACh^−1^), the mean value would likely remain at 1 but the standard deviation rises to 2.

The results in Figure 10B are illustrative to demonstrate the potential variability in infection risk that could result from exposures on a public transport system, but it is important to recognize that analysis of infection risk also needs to be interpreted in the context of the current status of the pandemic within a particular country or region. Emergence of more transmissible variants is already changing the exposure‐risk relationships, and it is likely that dose response will be specific to a particular variant.^43^ The risk of infection will also be substantially impacted by the vaccination status within a community. At the time of writing, 45 million people had received the first vaccine dose and 34 million the second dose in the UK, which will substantially reduce the likelihood of infection further than those illustrated here.

## 5 CONCLUSIONS

The TVC model presented here is a feasible approach for estimating the relative risks posed by different transmission routes and the influence of mitigation measures on public transport. The model is applied to a realistic subway train scenario to explore the influence of key parameters on the likely exposure to SARS‐CoV‐2 virus. For the parameters considered, the risk of exposure to the virus was predicted to be low through all routes of transmission. The highest modeled doses were to a small proportion of people in close proximity to an infected person, which is through a combination of aerosol inhalation and direct droplet deposition. Simulations predict that a small proportion of people may also receive a high fomite dose if they touch highly contaminated surfaces. Long‐range airborne exposure was generally predicted to be lower due to the short journey times and high ventilation rate, but was more homogeneous and dominates the median dose. This illustrates how a small aerosol can more readily reach multiple people, while close‐range and fomite exposures depend on chance encounters. The simulations suggested that prevalence of the virus within those traveling had the greatest influence on risk of transmission, and that social distancing (via loading) and mask wearing were both effective at reducing the average dose and the outliers. Ventilation influenced the median dose but had less effect on outliers which are dominated by the close‐range and fomite exposure.

We have shown the potential of QMRA‐based approaches to estimate exposure through different routes in public transport settings. The approach can be generalized to other public transport scenarios through appropriate information on vehicle geometries, ventilation, passenger loading, and touch surfaces. We have demonstrated how the model can be applied with a dose‐response function in order to translate exposure to infection risk; however, further data are needed on the dose response for the SARS‐CoV‐2 virus before this can be done with confidence. It will also be important to include the effects of vaccination in the future models such as this, considering the effects of the vaccine on the likelihood of an infectious traveler, the viral load in the event that the infectious person is vaccinated, and the dose response in the susceptible population.

## CONFLICTS OF INTEREST

None to declare.

## AUTHOR CONTRIBUTIONS

MLG, STP, and CJN lead the concept. DM, MFK, JN, JRG, and IH supported the conceptualization. MFK and STP lead data curation. DM, JN, JRD, and HC supported data curation. DM, MFK, MLG, and STP lead on the formal analysis. UD, IH, and CJN supported the formal analysis. DM, MFK, IH, STP, MLG, and CJN obtained funding. DM and STP lead on investigation. MFK, JN, JRD, UD, IH, and MLG supported the investigation. DM and STP lead on methodology. MFK, JN, JRD, UD, IH, MLG, and CJN supported methodology. MLG and STP lead on project admin. DM, JRD, and CJN supported project admin. DM, JN, JRD, and STP lead on software development. MFK lead on visualization with support from DM, JRD, JN, MLG, STP, and CJN. MLG and MFK wrote the manuscript. All other authors edited and reviewed the manuscript.

### PEER REVIEW

The peer review history for this article is available at https://publons.com/publon/10.1111/ina.12976.

## Supporting information

Supplementary Material
