# Supplementary material for: Modeling the factors that influence exposure to SARS‐CoV‐2 on a subway train carriage
Source: Indoor Air. 2022 Feb 8;32(2):e12976. doi: 10.1111/ina.12976 (PMC9111599; doi:10.1111/ina.12976)
Supplement: Supplementary file 1 — Supplementary Material [file INA-32-0-s001.pdf]

Modelling the factors that influence exposure to SARS-CoV-2 on a subway train carriage

Supplementary Material

Daniel Miller[†,a], Marco-Felipe King[†,b], James Nally[a], Joseph R Drodge[a], Gary I Reeves[a], Andrew M Bate[b], Henry Cooper[a], Ursula Dalrymple[a], Ian Hall[d], Martín López-García[#,c], Simon T Parker[#,a], Catherine J Noakes[#,b,*]

[a]*Defence Science and Technology Laboratory, Salisbury, SP40JQ, UK*
[b]*School of Civil Engineering, University of Leeds, Woodhouse Lane, Leeds, LS29JT, UK*
[c]*School of Mathematics, University of Leeds, Woodhouse Lane, Leeds, LS29JT, UK*
[d]*Department of Mathematics, University of Manchester, Oxford Road, Manchester, M139PL, UK*

[†] *These authors share first authorship.*
[#] *These authors share senior authorship.*

Abstract

In this supplementary material, we provide details on how the representative carriage is chosen when simulating passengers trips and estimating exposure to SARS-CoV-2 within the TVC model. We also show how passengers are allocated within 2m of an infected passenger during their journey, and give an overview of the method used in order to adjust the surface area in 0-1m and 1-2m of the infected passenger to account for possible positions of this passenger within the carriage. We estimate the surface area within the carriage as a whole and the region of the carriage within 2m of an infectious passenger. Finally, precise details on the implementation of the different droplet models and droplet evaporation calculations are provided, and a comprehensive list of parameter values within the TVC model is given.

1. Representative carriage selection

Instead of considering different carriage journeys as a stochastic element in the TVC model, which would be computationally prohibitive, a single carriage was selected, to represent an *average* or *representative* behaviour, for our numerical results. This representative carriage is depicted as a solid black line in Figure 2 in the main paper. In particular, the following approach was used to choose this representative carriage journey:

*Corresponding author, c.j.noakes@leeds.ac.uk

- Only southbound (SB) routes were considered to represent travel into the city.
- The total number of passengers carried over the trip in the representative carriage must be within 10% of the mean across all the carriages on the SB route.
- The integrated occupancy over all stops must be with 10% of the mean over all carriages on the SB route.
- We compare the boarding numbers at each station (normalised by total passengers on the journey) to the averages amongst the SB carriages, and select the carriage with the lowest error via an R^2 value.
- We visually check the route against occupancy patterns, and against stops travelled.
- We check linearity of carriage at different loading percentages is sufficient. In particular, the loading percentages for the selected carriage were found to be linear with an R2 value of 0.97.

2. Process of allocating passengers to be within 2m of an infectious passenger

In order to calculate a passenger's exposure as a result of being within 2m of an infectious passenger, a method for allocating passengers within the carriage is needed. This process occurs every time the passengers board and alight in each station, and is as follows:

1. When passengers board, the number of passengers on board, N_P is calculated. Passengers are initially assumed to be uniformly spread across the carriage, and classified to be within 0-1m, 1-2m or further than 2m away from infectious passengers.
2. The proportion of carriage floor area within 0-1m (A_{01} $[m^2]$) and 1-2m (A_{12} $[m^2]$) of an infectious passenger, and the total carriage floor surface area (A_F $[m^2]$) are used together with the number of passengers on board N_P to generate a target (rounded to an integer) number of passengers within 0-1m, T_{01}, and 1-2m, T_{02}, of an infectious passenger, as follows:
$$T_{01} = N_P \frac{A_{01}}{A_F}, \quad T_{12} = N_P \frac{A_{12}}{A_F}.$$
3. If the number of passengers initially allocated in 0-1m of an infectious passenger is less than T_{01} then, if possible, passengers are moved from the 1-2m region into the 0-1m region. If this is not possible then they are taken at random from the rest of the carriage.

4. If the number of passengers initially allocated in the 1-2m region is less than T_{12} then the spaces are filled by passengers from the rest of the carriage.
5. If the number of passengers within 0-1m of the infectious passenger is greater than T_{01} then the excess passengers are moved to the 1-2m region.
6. If the number of passengers in the 1-2m region is greater than T_{12} then passengers are moved out of the region into the rest of the carriage. Passengers moved into this region within the previous step are chosen last from the list of passengers eligible to be moved out of the region.

3. Adjustment of proportion of carriage within 2m of an infectious passenger

Here, we give an overview of the method used in order to adjust the surface area and available volume in 0-1m and 1-2m of the infectious passenger to account for possible positions of this passenger within the carriage; see Figure 1 which has been generated from available information in [1]. To begin, a rectangular grid is generated with the same dimensions as the width and length of the carriage. At each grid point, a random sample is generated from a uniform distribution for a 1m disc and for an annulus between 1m and 2m radii, see Figure 2. Then, the number of points that lie inside the carriage is counted and divided by the total number of points to yield a proportion of the disc/annulus that lies inside the carriage at a given point within the carriage. The proportion of the disc and the proportion of the annulus at a given point will be linked (Figure 1) and it is therefore necessary to consider what the proportion at 1-2m is, given the proportion at 0-1m.

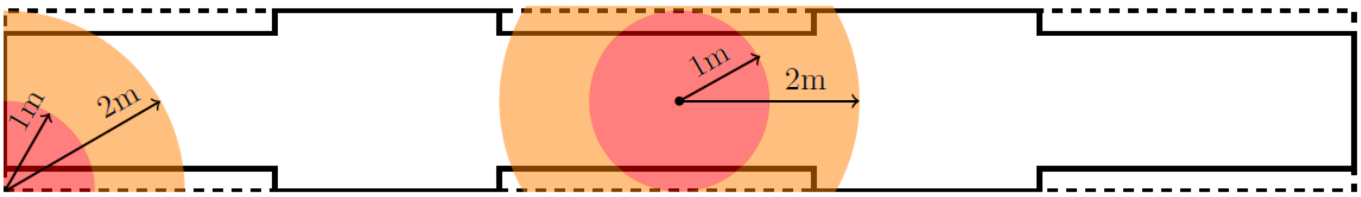

Figure 1: Depiction of 0-1m and 1-2m regions for different potential locations of the infectious passenger within the subway train carriage.

The array of proportions for 0-1m is then sorted from smallest to largest with the associated array of 1-2m values sorted according to the 0-1m value at that carriage position. The 0-1m values are then binned, the number of values in each bin counted and divided by the total number of points to generate a probability of the 0-1m value lying in that bin. An array of bin midpoints is also generated. We then

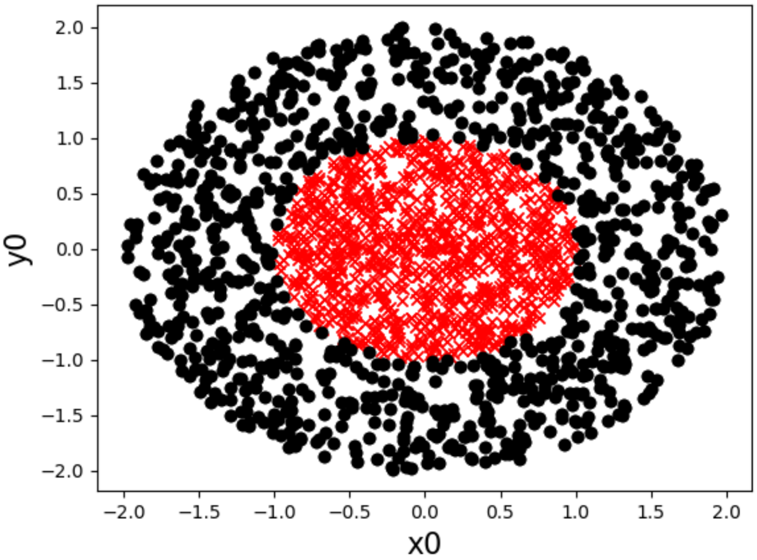

Figure 2: Uniform distribution of points within disc and annulus.

generate a sample of 0-1m carriage proportions (from the bin midpoints) with a weighting dictated by the probability of a value lying in that bin.

A probability distribution for the 1-2m values is then generated as follows. The 1-2m values for a given 0-1m bin are binned using the same bin edges and widths as the 0-1m bins. As before, the number of values in each bin is counted and used to generate the probability of the 1-2m value lying in a given bin. These can then be used in conjunction with the bin midpoints to generate a random sample of 1-2m values if the 0-1m lies in the associated bin. This means that there are as many 1-2m probability distributions as there are 0-1m bins.

For each passenger that boards the carriage, instead of estimating their 0-1m and 1-2m proportions (i.e., adjusted surface areas) by randomly allocating the passenger within the carriage and following the approach above, their 0-1m and 1-2m proportions are directly sampled from the distributions computed *a priori* as described above. This decreases computational cost of running stochastic simulations of the carriage trip. In Figure 3 we carry out some numerical experiments to confirm that the resulting 0-1m and 1-2m proportions for any passenger are appropriately estimated in this way. In this figure, green points result from randomly selecting a position in the carriage, red from regularly separated points (at 0.1m distance) organised in a rectangular grid, and grey are points sampled from our distributions with their size linearly related to the number of points overlapping at a given point on the plot. As one can notice, and for a large number of estimates, these approaches are in agreement.

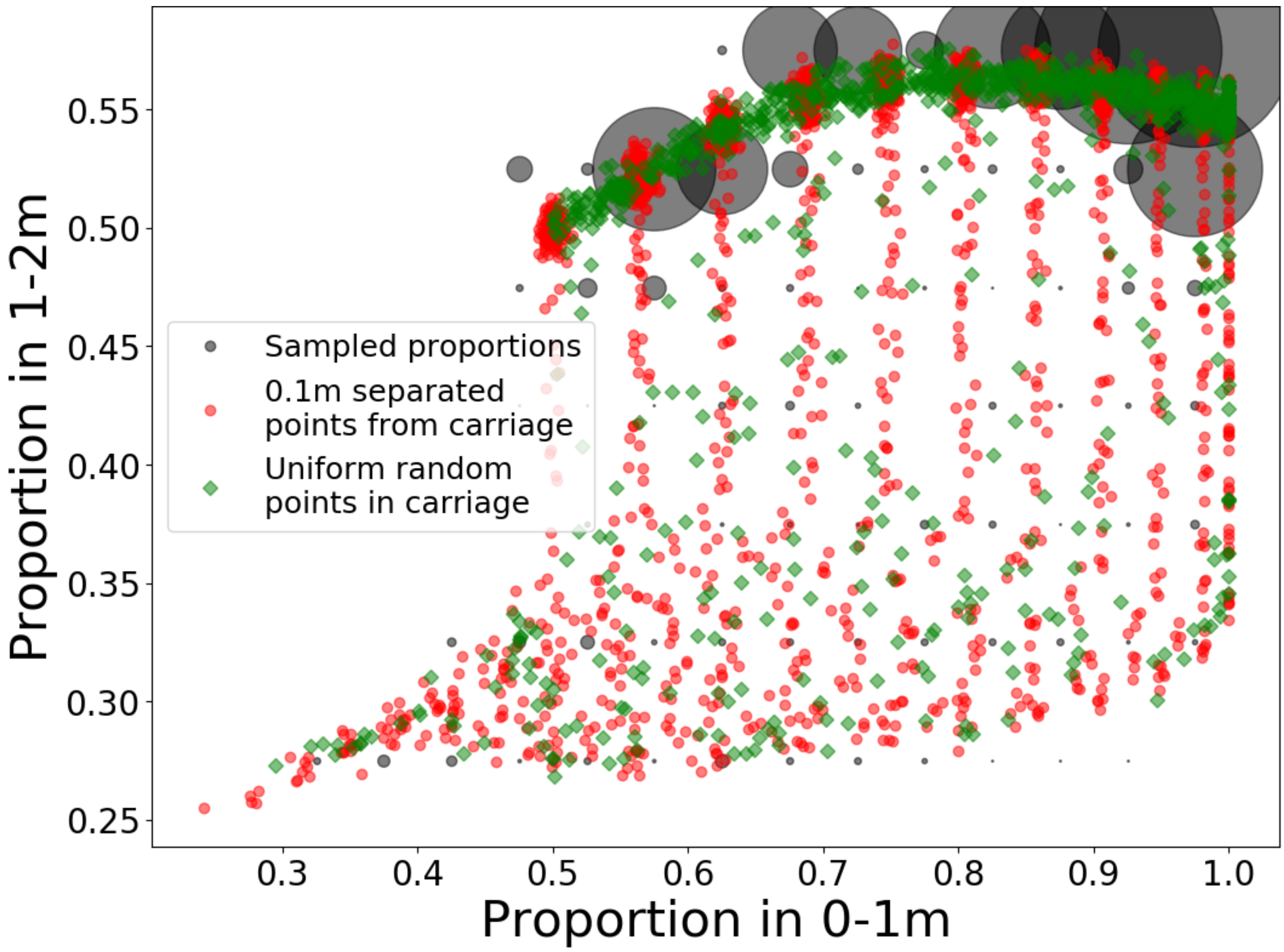

Figure 3: Position of sampled values relative to proportions directly from the carriage.

4. Surface area within the carriage

To calculate the fraction of deposited droplets that deposits onto mucosal membranes, it is required to obtain an estimate of the surface area (SA) within the carriage as a whole and the region of the carriage within 2m of an infectious passenger.

The total surface area within the carriage has the following components: the carriage floor surface area, the ceiling surface area, the four walls areas, the number of internal surfaces multiplied by their surface area (see Table 2 in this Supplementary Material), the number of passengers on board at any given time multiplied by their surface area (see Table 2 in this Supplementary Material). On the other hand, the components of the surface area within 2m of an infectious passenger are: the floor area within 2m (computed following methodology in Section 3 in this Supplementary Material), the number of surfaces within 2m (estimated by multiplying the total number of surfaces in the carriage by the

fraction of floor area within 2m) multiplied by their surface area, the number of passengers within 2m multiplied by their surface area, and an estimate of the wall surface area within 2m of the passenger ($13.05m^3$, estimated by performing Monte Carlo simulations of the positions within the carriage and calculating the mean).

5. Droplet size distribution

There is no current consensus on which droplet size distribution best fits human behaviour for the activities which are of most interest (coughing, speaking, breathing). As such, choice of droplet size distribution varies significantly within the literature. One common choice is to use the data given by Duguid [2] or Loudon and Roberts [3, 4], with the data on number of droplets and droplet diameter for coughing therein being widely referenced. Another approach is to use the bronchiolar/laryngeal/oral (BLO) model which is used within [5] and which models the number concentration of droplets in a given size bin via a lognormal distribution. Another common choice is that proposed by Nicas et al. [4, 6], which is to use a probability distribution function (PDF) generated by summing two lognormal distributions. This approach is not used here but is used in, for example, [7].

We have implemented approaches based on droplet data [2, 3] and the BLO model [5] into the TVC model, and explore their impact on our exposure estimates. The main model variables/parameters related to this are *dropletModel*, *exhaleType*, *largeDropletMin* and *largeDropletMax*. If one chooses "Duguid" as the *dropletModel* parameter, the TVC model considers the midpoints of the droplet diameter from the cough data within [2], and the number of droplets in the bin along with the number of coughs per second and the viral load to generate a list of (wet) droplet diameters and an associated source term Ω_j $[PFU \cdot s^{-1}]$ for each droplet diameter, for droplet sizes $j = 1, \ldots, M$. The *largeDropletMin* and *largeDropletMax* parameters then provide upper and lower bounds on which of these droplet sizes are considered as "large droplets" in the model (this is the same for all of the distributions). The same process occurs if "LR" is selected as the dropletModel. In this case the data is taken from the paper [4] but originally reported in [3]. We follow the same procedure as [4] and double the droplet diameters as it was assumed that the droplet sizes reported in [3] were evaporated sizes.

If "BLO" is selected as the *dropletType* then the BLO model [5] is used. This is implemented using the approach within [8]. Given a bin j, its minimum and maximum values ($x_{j_{start}}$ and $x_{j_{stop}}$) and its midpoint (x_j), the number concentration (number of droplets per m^3) inside the bin is given by the

following sum of lognormal distributions:

$$\begin{aligned}\frac{dCn_j}{dLogx_j} &= ln10\sum_{i=1}^{3}\left(\frac{Cn_i}{\sqrt{2\pi}ln(GSD_i)}\right)e^{-\frac{(ln(x_j)-ln(CMD_i))^2}{2\cdot(ln(GSD_i))^2}},\\ dLogx_j &= logx_{j_{stop}} - logx_{j_{start}},\end{aligned}$$

with the parameters in Table 1.

	Parameter	**Coughing**	**Speaking**	**Breathing**
	Cn_1 $[m^{-3}]$	9.03E4	5.4E4	5.4E4
**B mode**	CMD_1 $[m]$	1.57E-6	1.61E-6	1.61E-6
	GSD_1 [-]	1.25	1.30	1.3
	Cn_2 $[m^{-3}]$	1.42E5	6.8E4	N/A
**L mode**	CMD_2 $[m]$	1.60E-6	2.40E-6	N/A
	GSD_2 [-]	1.68	1.66	N/A
	Cn_3 $[m^{-3}]$	1.60E4	1.26E3	N/A
**O mode**	CMD_3 $[m]$	1.23E-4	1.44E-4	N/A
	GSD_3 [-]	1.84	1.80	N/A

Table 1: BLO model input parameters [5]. Breathing only uses the B mode.

By assuming an homogeneous viral load per unit volume across droplet sizes, and for a particular droplet size $j = 1, \ldots, M$, the volume concentration $[m^3 \cdot m^{-3}]$ is then calculated using the formula

$$dC_{vol_j} = \frac{4}{3}\cdot\pi\cdot\left(\frac{x_j}{2}\right)^3\cdot dCn_j,$$

and the source term $[PFU \cdot s^{-1}]$ for coughing is found via

$$\Omega_j = \frac{dC_{vol_j}\cdot V_{air}\cdot\omega}{T_c},$$

where ω is the viral load $[PFU \cdot m^{-3}]$, V_{air} is the volume of air exhaled during a cough $[m^3]$ and T_c is the time between coughs $[s^{-1}]$. For speaking and breathing the source term is given by

$$\Omega_j = dC_{vol_j}\cdot BR\cdot\omega,$$

where BR is the breathing rate $[m^3 \cdot s^{-1}]$.

Finally, within the TVC model individual passengers may or may not be wearing a mask. The percentage of passengers whom are wearing a mask is denoted by $Mask_{\%}$. This parameter's value is

between 0 and 100, with 100 denoting all passengers wearing masks and 0 indicating that no passengers are wearing masks. The impact of wearing a mask for infectious individuals is a reduction in their release of small aerosol and droplets. In particular, it is assumed that masks block all large droplets, while a 50% filtration efficacy is assumed for small aerosols [9, 10]. This filtration efficacy is also applied to reduce the exposure of susceptible passengers who are wearing a mask.

In Figures 4 and 5, we explore the impact that the interaction between the droplet model under consideration and the values of the key parameters varied in Section 3 within the main manuscript has on exposure. We note that Duguid's droplet model predicts consistently higher median values than any of the others, particularly when comparing *BLO breathing* or *coughing* model (e.g. Figure 4a). Duguid's droplet model also predicts a relatively larger contribution of the long range airborne route, although the highest doses are still predicted to occur via the close range and fomite routes. Interestingly, the *BLO speaking* typically predicts higher median values than the *BLO coughing* model whereas this trend is reversed when analysing the mean values. As a result, it suggests that there may be less opportunistic events when the infectious person is speaking rather than coughing, under these viral loads. However, we note here that while speaking happens in our model continuously during the infectious passenger trip, coughing is assumed to occur at a given frequency instead. It is to be expected that if an assumption about the fraction of the journey where the passenger is speaking was incorporated, this relationship could change. The effect of mask wearing compliance in Figure 5a shows how the variability in the predicted mean between droplet models is reduced to the same order of magnitude when all passengers comply with mask wearing at 100%.

6. Evaporation of respiratory droplets

Evaporation of respiratory droplets affects the resulting particle size. The act of drying is also believed to affect viral viability. The majority of the initial droplets produced dry rapidly and for the purposes of calculating deposition rates within the concentration and exposure calculations, it is assumed that all droplets rapidly dry to their final size. This is likely to slightly underestimate deposition rates for the largest droplet sizes. However, deposition rates for these sizes are high (even when dry) so this does not introduce a large source of error. The dry size is also used for any removal due to face coverings worn by exposed individuals, while wet droplet sizes are used for estimating source reduction by face coverings of infected individuals.

However, so as not to underestimate the effect of larger droplets, the loss of viability due to drying

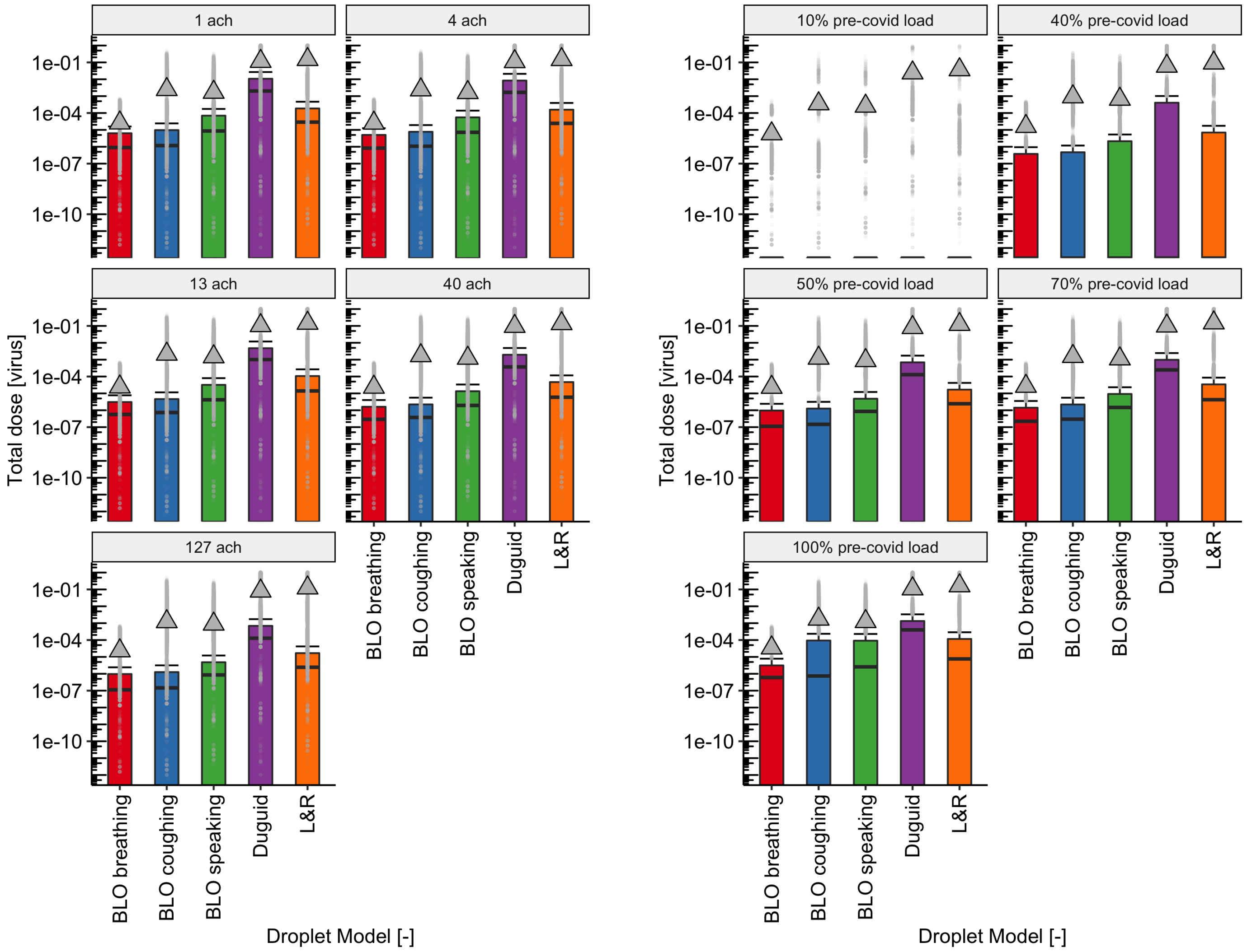

(a) Fresh-flow ventilation rate.

(b) Percentage loading with respect to pre-COVID-19.

Figure 4: Boxplots showing the effect of the five droplet models varying disease and individual parameters one at a time on total dose received.

is determined following the approach of [7]. In particular, we define an evaporation time

$$T_e \;=\; \beta \cdot r_0^2,$$

where β $[s \cdot m^{-2}]$ is a fitting parameter and r_0^2 is the wet droplet radius squared. We also define a travel time for a droplet to be

$$T_s \;=\; \frac{s}{v_m}$$

where s is the distance travelled and v_m the droplet speed $[m \cdot s^{-1}]$. The distance s takes the value 0.5m (T_{05}) or 1.5m (T_{15}) depending on the distance of the susceptible passenger to the infectious passenger

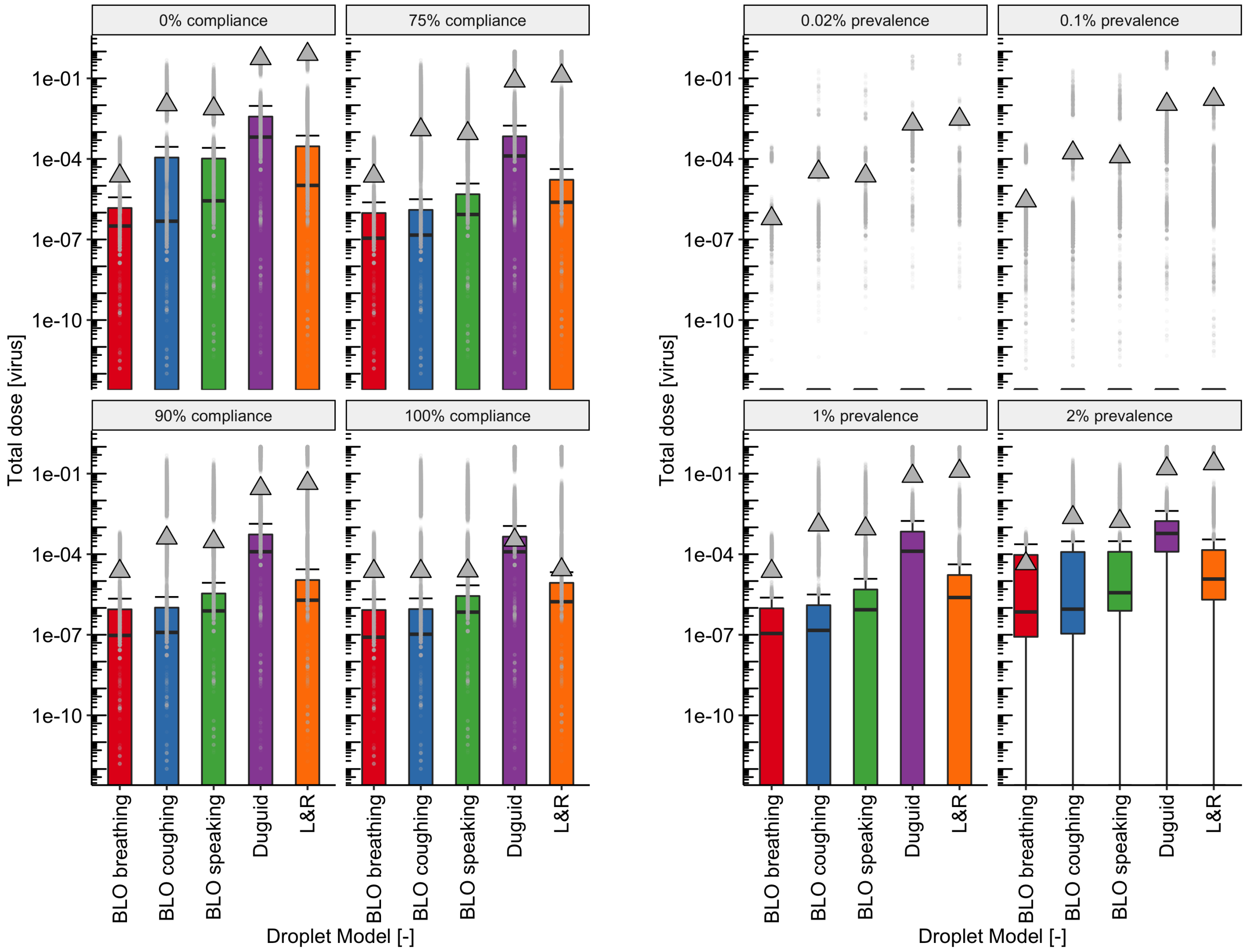

(a) Mask compliance percentage.

(b) Prevalence of disease within the population.

Figure 5: Boxplots showing the effect of the five droplet models varying disease and individual parameters one at a time on total dose received.

(either within 1m or 1-2m away). We then have the following scenarios for a droplet of a given wet size:

- If $T_e < T_{05}$ then the droplet has dried before reaching any passengers. In this scenario the close range exposure for all passengers within $2m$ is reduced by a factor of 4 to represent the loss of viability [7].
- If $T_{05} < T_e < T_{15}$ then passengers within $1m$ of the infectious passenger receive the full close range exposure but passengers within $1 - 2m$ have theirs reduced by a factor of 4.
- If $T_e > T_{15}$ then the droplet is assumed to still be wet for all passengers within $2m$ and they thus

receive the full exposure.

We note that the exposures and doses due to small aerosol are all multiplied by 0.25 before being used to calculate final exposure upon alighting as the drying time for the small aerosol is assumed to be smaller than the distance to all passengers.

Finally, a solid fraction, α, is defined as an input parameter which represents the proportion of the droplet volume that is made of solid material. A fully evaporated droplet thus has volume $\alpha \cdot V_0$, where V_0 is the volume of the wet droplet. Assuming the remaining solid following full evaporation is spherical gives the expression

$$r_1 = \alpha^{\frac{1}{3}} \cdot r_0$$

for the evaporated droplets radius. As discussed above, it is this radius which is used within the TVC model wherever calculations of deposition, filtration or protection of exposed individuals by masks require the use of a droplet radius, with the exception of source reduction for infected individuals due to mask wearing.

7. Parameter values

A comprehensive list of default parameter values is given in Table 2.

**Related Route**	**Parameter**	**Description**	**Units**	**Default value**	**Source**
General	ϕ	Prevalence; the proportion of passengers boarding who are infectious	-	Varied	-
General	ρ	System loading percentage	-	Varied	-
General	BR	Rate at which passenger breathes.	$m^3 \cdot s^{-1}$	$1.72454 \cdot 10^{-4}$	[11]
General	ω	The viral load of SARS-CoV-2 in respiratory fluid. Computed by dividing RNA copies per m^3 from [12] ($4.7 \cdot 10^{14}$ RNA/m^3) by number of RNA per PFU from [13] (130 RNA/PFU)	$PFU \cdot m^{-3}$	$3.61 \cdot 10^{12}$	[12, 13]
General	H	Height of the carriage	m	2.148	[1]
General	HSA	Human surface area. Used for total deposition surface area in carriage	m^2	1.75	[7]
General	$Mask_{\%}$	Percentage of passengers who wear masks	-	Varied	-

Close range	α	Fraction of the droplet volume which is solid	-	0.25^3	[7]
Close range	β	Fitting parameter which is used to calculate a droplet's evaporation time	$s \cdot m^{-2}$	$7 \cdot 10^8$	[7]
Close range	v_m	The droplet speed. Used to calculate how long a droplet takes to travel a fixed distance	$m \cdot s^{-1}$	0.1	[7]
Close range	$largeDropletMin$	The minimum droplet diameter considered for close range transmission as a "large" droplet	m	$2 \cdot 10^{-5}$	Assumed
Close range	$largeDropletMax$	The maximum droplet diameter considered for close range transmission as a "large" droplet	m	$2 \cdot 10^{-3}$	Assumed
Close range	V_{air}	The volume of air expelled during a cough. Only used with the BLO droplet model	m^3	$1.69 \cdot 10^{-3}$	[14]
Close range	k_d	Coefficient of particle deposition	$s^{-1} \cdot m^{-2}$	$3.89 \cdot 10^7$	[7]
Small aerosol	RF_{small}	Proportion of the small aerosol inhaled which is retained	-	0.8	[15]
Large aerosol	RF_{large}	Proportion of the large aerosol inhaled which is retained	-	0.6	[15]
Long range aerosol	V	Carriage volume	m^3	53.2	Based on dimensions within [1] and adapted to account for seating and occupants.
Long range aerosol	r_v	Fresh air flow rate in the carriage	$m^3 \cdot s^{-1}$	1.9	Based on passenger theoretical crush capacity within [1] and an assumed supply rate of 10 L/s/person.
Long range aerosol	r_i	Virus decay rate in aerosol, fractional loss per second of virus in the air due to inactivation	s^{-1}	$3.78 \cdot 10^{-4}$	[16]

Long range aerosol	r_d	Deposition rate in carriage. Assuming a factor of four for the drying ratio, using cough source data from [2]. Calculations performed using model described in [7] from [17]	s^{-1}	$1.82 \cdot 10^{-4}$	[2, 7, 17]
Surface contact	N_T	Total number of surfaces in the carriage. This consists of 57 touch points on poles and door handles, 25 touch points on chair handles and 32 touch points on horizontal railings. Surfaces are assumed to be hard and non-porous.	-	114	[1]
Surface contact	N_{HS}	Number of surface touches when boarding and alighting	-	3	Assumed
Surface contact	τ_{HS}	Transfer efficiency from hand to surface	-	0.27	[7]
Surface contact	τ_{SH}	Transfer efficiency from surface to hand	-	0.29	[7]
Surface contact	τ_{HM}	Transfer efficiency from hand to mucous membrane on face (eyes, mouth, lips)	-	0.36	[7]
Surface contact	δ_H	Inactivation rate on hands	s^{-1}	$5.5 \cdot 10^{-5}$	[18]
Surface contact	δ_S	Inactivation rate on surface	s^{-1}	$6.22 \cdot 10^{-5}$	[19]
Surface contact	C_{H0}	Initial concentration on infected passenger hands	$PFU \cdot m^{-2}$	1500	[20]
Surface contact	A_H	Area of full hand	m^2	0.042	[21]
Surface contact	A_P	Area of front of hand	m^2	0.02016	[21]
Surface contact	A_M	Area of mucous membranes (lips+eyes+mouth)	m^2	$1 \cdot 10^{-3}$	[7]
Surface contact	A_S	Area of surface, equal for all surfaces	m^2	0.04	Assumed. Representative surface is a circular pole of diameter 25mm and length 50cm, leading to $0.04m^2$
Surface contact	A_{HM}	Area of hand-membrane contact area	m^2	$1 \cdot 10^{-4}$	[7]

Surface contact	A_{HS}	Area of hand-surface contact area. Obtained by multiplying A_H by $HCF = 0.148$ (fractional surface area; fraction of hand touching the surface), as measured in [22]	m^2	$6.216 \cdot 10^{-3}$	[22]
Surface contact	ξ_m	Touches to mucous membranes per second	s^{-1}	$1.389 \cdot 10^{-3}$ (5 per hour)	[7]
Surface contact	T_a	Time after alighting that the passengers face touching contamination transfer continues before hand washing	s	900	Assumed. Represents a situation where passengers do not wash their hands during the first $15min$ after alighting.
Surface contact	T_c	Time between coughs	s	60 (1 cough per minute)	Assumed
Surface contact	A_{proj}	Projected area of surface (equal to length $\times$ width of the representative cylindrical surface)	m^2	0.0125	Assumed
Surface contact	A_{depo}	Spread of droplets in the air within the horizontal and vertical directions at a distance $0.5m$ away from the coughing passenger	m^2	0.25	Assumed

Table 2: Default parameter values in the TVC model.

References

[1] Transport for London (2011) “2009 Tube Stock” Transport for London FOI request [Online], https://www.whatdotheyknow.com/request/247718/response/615841/attach/7/09%20Stock.pdf?cookie_passthrough=1.

[2] J. P. Duguid, The size and the duration of air-carriage of respiratory droplets and droplet-nuclei, Journal of Hygiene 44 (6) (1946) 471–479. doi:10.1017/S0022172400019288.

[3] R. G. Loudon, R. M. Roberts, Droplet expulsion from the respiratory tract, American Review of Respiratory Disease 95 (3) (1967) 435–442.

[4] M. Nicas, W. W. Nazaroff, A. Hubbard, Toward Understanding the Risk of Secondary Airborne Infection: Emission of Respirable Pathogens, Journal of Occupational and Environmental Hygiene 2 (3) (2005) 143–154. doi:10.1080/15459620590918466.
URL https://doi.org/10.1080/15459620590918466

[5] G. Johnson, L. Morawska, Z. Ristovski, M. Hargreaves, K. Mengersen, C. Chao, M. Wan, Y. Li, X. Xie, D. Katoshevski, S. Corbett, Modality of human expired aerosol size distributions, Journal of Aerosol Science 42 (12) (2011)

839 – 851. doi:https://doi.org/10.1016/j.jaerosci.2011.07.009.
URL http://www.sciencedirect.com/science/article/pii/S0021850211001200

[6] M. P. Atkinson, L. M. Wein, Quantifying the routes of transmission for pandemic influenza, Bulletin of mathematical biology 70 (3) (2008) 820–867.

[7] H. Lei, Y. Li, S. Xiao, C.-H. Lin, S. L. Norris, D. Wei, Z. Hu, S. Ji, Routes of transmission of influenza A H1N1, SARS CoV, and norovirus in air cabin: comparative analyses, Indoor Air 28 (3) (2018) 394–403.

[8] A. Hill, M. Testolin, R. Brown, M. Jamriska, A Contaminated Surface Pickup Model and its Application to COVID-19 Infection, Tech. rep., DST-Group-RR-0462. Australian Government: Department of Defence Science and Technology (2020).

[9] The Royal Society, Face masks and coverings for the general public: Behavioural knowledge, effectiveness of cloth coverings and public messaging, Tech. rep. (June 2020).

[10] A. Konda, A. Prakash, G. A. Moss, M. Schmoldt, G. D. Grant, S. Guha, Aerosol filtration efficiency of common fabrics used in respiratory cloth masks, ACS nano 14 (5) (2020) 6339–6347.

[11] B. S. Binkowitz, D. Wartenberg, Disparity in quantitative risk assessment: A review of input distributions, Risk Analysis 21 (1) (2001) 75–90.

[12] T. Edwards, V. S. Santos, A. L. Wilson, A. I. Cubas-Atienzar, K. Kontogianni, C. T. Williams, E. R. Adams, L. E. Cuevas, Variation of sars-cov-2 viral loads by sample type, disease severity and time: a systematic review, medRxiv (2020).

[13] G. Buonanno, L. Morawska, L. Stabile, Quantitative assessment of the risk of airborne transmission of sars-cov-2 infection: prospective and retrospective applications, Environment International 145 (2020) 106112.

[14] J. Lee, D. Yoo, S. Ryu, S. Ham, K. Lee, M. Yeo, K. Min, C. Yoon, et al., Quantity, size distribution, and characteristics of cough-generated aerosol produced by patients with an upper respiratory tract infection, Aerosol and Air Quality Research 19 (4) (2019) 840–853.

[15] International Organization for Standardization (2012) "ISO 13138:2012" 13.040.01 Air Quality in General., https://www.iso.org/standard/53331.html, accessed: 27-04-2021.

[16] S. J. Smither, L. S. Eastaugh, J. S. Findlay, M. S. Lever, Experimental aerosol survival of sars-cov-2 in artificial saliva and tissue culture media at medium and high humidity, Emerging microbes & infections 9 (1) (2020) 1415–1417.

[17] T. L. Thatcher, A. C. Lai, R. Moreno-Jackson, R. G. Sextro, W. W. Nazaroff, Effects of room furnishings and air speed on particle deposition rates indoors, Atmospheric environment 36 (11) (2002) 1811–1819.

[18] D. E. Harbourt, A. D. Haddow, A. E. Piper, H. Bloomfield, B. J. Kearney, D. Fetterer, K. Gibson, T. Minogue, Modeling the stability of severe acute respiratory syndrome coronavirus 2 (sars-cov-2) on skin, currency, and clothing, PLoS neglected tropical diseases 14 (11) (2020) e0008831.

[19] M. J. Matson, C. K. Yinda, S. N. Seifert, T. Bushmaker, R. J. Fischer, N. van Doremalen, J. O. Lloyd-Smith, V. J. Munster, Effect of environmental conditions on sars-cov-2 stability in human nasal mucus and sputum, Emerging infectious diseases 26 (9) (2020) 2276.

[20] NERVTAG-EMG SAGE paper on hand hygiene, https://assets.publishing.service.gov.uk/government/uploads/system/uploads/attachment_data/file/897598/S0574_NERVTAG-EMG_paper_-_hand_hygiene_010720_Redacted.pdf, accessed: 07-05-2021.

[21] J.-Y. Lee, J.-W. Choi, H. Kim, Determination of hand surface area by sex and body shape using alginate, Journal of Physiological Anthropology 26 (4) (2007) 475–483.
[22] W. AuYeung, R. A. Canales, J. O. Leckie, The fraction of total hand surface area involved in young children's outdoor hand-to-object contacts, Environmental research 108 (3) (2008) 294–299.
